# Multicolor Melting Curve Analysis-Based Multilocus Melt Typing of *Vibrio parahaemolyticus*

**DOI:** 10.1371/journal.pone.0136998

**Published:** 2015-09-14

**Authors:** Ran Liu, Zanzan Liu, Ye Xu, Yiqun Liao, Qinghua Hu, Jianwei Huang, Xiaolu Shi, Yinghui Li, Jianjun Niu, Qingge Li

**Affiliations:** 1 Engineering Research Centre of Molecular Diagnostics, Ministry of Education, State Key Laboratory of Cellular Stress Biology, School of Life Sciences, Xiamen University, Xiamen, Fujian, China; 2 State Key Laboratory of Molecular Vaccinology and Molecular Diagnostic, School of Public Health, Xiamen University, Xiamen, Fujian, China; 3 Shenzhen Major Infectious Disease Control Key Laboratory, Shenzhen Centre for Disease Control and Prevention, Shenzhen, Guangdong, China; 4 School of Life Sciences, Shenzhen University, Shenzhen, Guangdong, China; 5 Xiamen Center for Disease Control and Prevention, Xiamen, Fujian, China; 6 Zhongshan Hospital of Xiamen, Xiamen University, Xiamen, Fujian, China; 7 Shenzhen Research Institute of Xiamen University, Shenzhen, Guangdong, China; University of Ulster, UNITED KINGDOM

## Abstract

*Vibrio parahaemolyticus* is the leading cause of seafood-borne gastroenteritis outbreaks. To track the source of these diseases in a timely manner, a high throughput typing method is critical. We hereby describe a novel genotyping method for *V*. *parahaemolyticus*, termed multilocus melt typing (MLMT), based on multilocus sequence typing (MLST). MLMT utilizes melting curve analysis to interrogate the allelic types of a set of informative single nucleotide polymorphisms (SNPs) derived from the housekeeping genes used in MLST. For each SNP, one allelic type generates distinct T_*m*_ values, which are converted into a binary code. Multiple SNPs thus generate a series of binary codes, forming a melt type (MT) corresponding with a sequence type (ST) of MLST. Using a set of 12 SNPs, the MLMT scheme could resolve 218 *V*.*parahaemolyticus* isolates into 50 MTs corresponding with 56 STs. The discriminatory power of MLMT and MLST was similar with Simpson’s index of diversity of 0.638 and 0.646, respectively. The global (adjusted Rand index = 0.982) and directional congruence (adjusted Wallace coefficient, MT→ST = 0.965; ST→MT = 1.000) between the two typing approaches was high. The entire procedure of MLMT could be finished within 3 h with negligible hands on time in a real-time PCR machine. We conclude that MLMT provides a reliable and efficient approach for *V*. *parahaemolyticus* genotyping and might also find use in other pathogens.

## Introduction

*Vibrio parahaemolyticus* is a gram-negative, halophilic marine bacterium, which can cause gastroenteritis through consumption of raw or undercooked seafood [1]. It is globally disseminated in estuarine areas and epidemic outbreaks are usually reported in coastal countries and regions [2–7]. At the moment, *V*. *parahaemolyticus* has been recognized as the leading cause of seafood-borne gastroenteritis and has increasingly raised public health concerns [4]. The frequent incidence of infections renders significance to surveillance and epidemiological investigation of this pathogen. An easy to use, cost-effective, and discriminatory typing scheme is therefore warranted for *V*. *parahaemolyticus*.

Multilocus sequence typing (MLST) is a genotyping approach based on comparative sequences from housekeeping genes to analyze the population structure of microbial isolates. On account of the reproducibility and portability of data, it is regarded as a preferred choice for investigation of population structure of *V*. *parahaemolyticus* [8–10]. Nevertheless, MLST is relatively expensive and time-consuming owing to the sequencing analysis of seven fragments of housekeeping genes for each isolate. Alternative simplified MLST-derivative methods have been developed. Among them, one approach is to choose a set of single nucleotide polymorphisms (SNPs) of high Simpson’s index of diversity (SID). These informative SNP sets are then interrogated by allele-specific real-time PCR [11–14] or high resolution melting [15–18].This approach has shown great potential as a rapid and cost-effective genotyping tool to complement MLST in the epidemiological study of various pathogens. However, many reactions have to be employed when multiple SNPs are detected, thus lowering the overall throughput of these methods.

We previously established a multicolor melting curve analysis to detect the genotype of mutations using dual-labeled, self-quenched probes on a real-time PCR platform [19]. This melting curve analysis approach proved to be a reliable and rapid tool in the detection of multiple mutations or SNPs in a single reaction tube [20–22]. In this study we sought to use this approach to establish a new genotyping scheme of high throughput. This scheme, termed multilocus melt typing (MLMT), could provide melting temperature (T_*m*_) values for the allelic types of the SNPs. The T_*m*_ values were converted into binary codes to generate a melt type (MT), which could be defined in correspondence with sequence type (ST) of MLST. MLMT provided digital results, ensuring the portability of data, which is important for data storage and transfer. As a model, *V*. *parahaemolyticus* was genotyped using a 12-SNP scheme. By analyzing 218 isolates of *V*. *parahaemolyticus*, we concluded that MLMT could provide rapid and discriminatory typing with high throughput and cost-effectiveness.

## Methods

### Bacterial isolates and genomic DNA preparation

One hundred and thirty-five clinical isolates were from Shenzhen Center of Disease Control and Prevention (Shenzhen CDC, Shenzhen, China), and 83 isolates, including 49 clinical isolates and 34 environmental isolates, were provided by Xiamen CDC (Xiamen, China). Ten isolates with known STs (ST-3, 199, 332, 345) were from our laboratory collection and were used to establish MLMT. Genomic DNA was prepared by using the AxyPrep^TM^ Bacterial Genomic DNA Miniprep Kit (Axygen Biosciences). Isolated DNA was quantified by a Nanodrop spectrophotometer (Nanodrop Products, Wilmington, Delaware, US). Prepared genomic DNA was stored at -20°C before use.

### Ethics Statement

All clinical isolates were collected for routine diagnostics by Shenzhen CDC and Xiamen CDC and were supplied for this study as coded specimens without any patient information or identifiers that could be used to decode patient information. The current study was thus exempted from ethical approval by the Ethics Committee on Human Studies in Xiamen University.

### SNPs selection

Sequences of 798 ST profiles including seven housekeeping genes (*recA*, *dnaE*, *gyrB*, *dtdS*, *pntA*, *pyrC* and *tnaA*) were retrieved from the *V*. *parahaemolyticus* MLST website (http://pubmlst.org/vparahaemolyticus/, accessed 21 June, 2013). The seven genes were aligned into concatenated sequences (3682 bps). The sequences were analyzed by Minimum SNPs software [14] to select a SNP set of high SID. “Simpson’s Index (0.0–1.0)” in the Allele Identification Parameters panel started at a low SID value of 0.90, and was raised up to a value of 0.9999. Dimorphic SNPs were considered as candidates for better discrimination by probes. The candidate SNPs were further evaluated regarding the conservation of the neighboring nucleotides by sequence alignment. The typing results were confirmed by the “Working Backwards Method” in the software, which can define STs based on the SNP profiles.

### Probes design

Dual-labeled probes for the detection of selected SNPs are listed in S1 Table. Probes were designed using Biophysics Version 1.00 (http://biophysics.idtdna.com). When needed, locked nucleic acids (LNAs) were introduced on the SNP positions to increase T_*m*_ difference (ΔT_*m*_) [23] between matched and mismatched targets. Inosine (I) and mismatch bases were introduced to neutralize the influence from non-target SNPs.

Three types of T_*m*_ were used for confirmation of the probe design: i) The *in silico* T_*m*_ for probe:target were obtained by Biophysics under the conditions below: 200 nM probe, 200 nM target, 50 mM Na^+^, K^+^, 3 mM Mg^2+^ and 200 mM deoxyribonucleoside triphosphates (dNTPs). ii) Hybridization T_*m*_s were obtained by using synthesized target oligonucleotides hybridized with probes in a thermal denaturation experiment: each 25-μL solution contained 0.2 μM probe, 0.4 μM target oligonucleotides, 67 mM Tris-HCl (pH 8.8), 16 mM (NH_4_)_2_SO_4_, 0.01% (W/V) Tween-20 and 2.5 mM MgCl_2_. The thermal denaturation procedure started at 95°C for 1 min, 35°C for 2 min, followed by melting analysis ramping from 30°C to 85°C in 0.5°C increments. Probes were synthesized and purified by Sangon (Shanghai, China). iii) The PCR T_*m*_s were obtained using isolates of known MLST types. For those isolates not found in our collection, artificial plasmids containing the amplification region were constructed to simulate real isolates. The obtained T_*m*_s from post-PCR were used to determine the critical T_*m*_s, by which the binary code of the allelic types was defined. The PCR conditions were provided in “MLMT procedure” below.

### Primers for MLST and MLMT

MLST primers are listed in S2 Table. A portion of MLST primers were redesigned for efficient amplification, which encompass the original MLST primers [8]. Primer design was based on the complete sequences of *V*. *parahaemolyticus* chromosome I and II in NCBI: RIMD 2210633 (BA000031, BA000032) [24], O1:K33 str. CDC_K4557 (CP006007, CP006008), BB22OP (CP003972, CP003973) [25], O1:Kuk str. FDA_R31 (CP006004, CP006005). MLMT primers are listed in S3 Table, with the size of amplicons from 113~248 bps. Primers were designed by using Premier Primer 5.0 (Premier Biosoft International, Palo Alto, CA), and Oligo 6.0 (AVG Technologies Inc., Chelmsford, MA). Primers were synthesized and purified by Sangon.

### MLST procedure

PCR was conducted under the following thermocycling conditions: 95°C for 5 min, 15 cycles of touchdown PCR (95°C for 10 s, 69°C with 1°C/cycle decrease for 20 s, and 72°C for 20 s) and 35 cycles of 95°C for 10 s, 55°C for 20 s, and 72°C for 20 s. PCR products were resolved by gel electrophoresis. Sequencing was performed by BGI (Shenzhen, China). Sequences were analyzed online (http://pubmlst.org/vparahaemolyticus/) to assign allele numbers and define STs. New sequences for alleles and new ST profiles were submitted to the *V*. *parahaemolyticus* MLST database. The clonal complexes(CCs) of *V*. *parahaemolyticus* were analyzed by goeBURST [26] of Phyloviz software (http://www.phyloviz.net) [27]. Those STs that share identical alleles at six of the seven MLST loci with at least one other ST were classified as one CC [28].

### MLMT procedure

**Fig 1:**
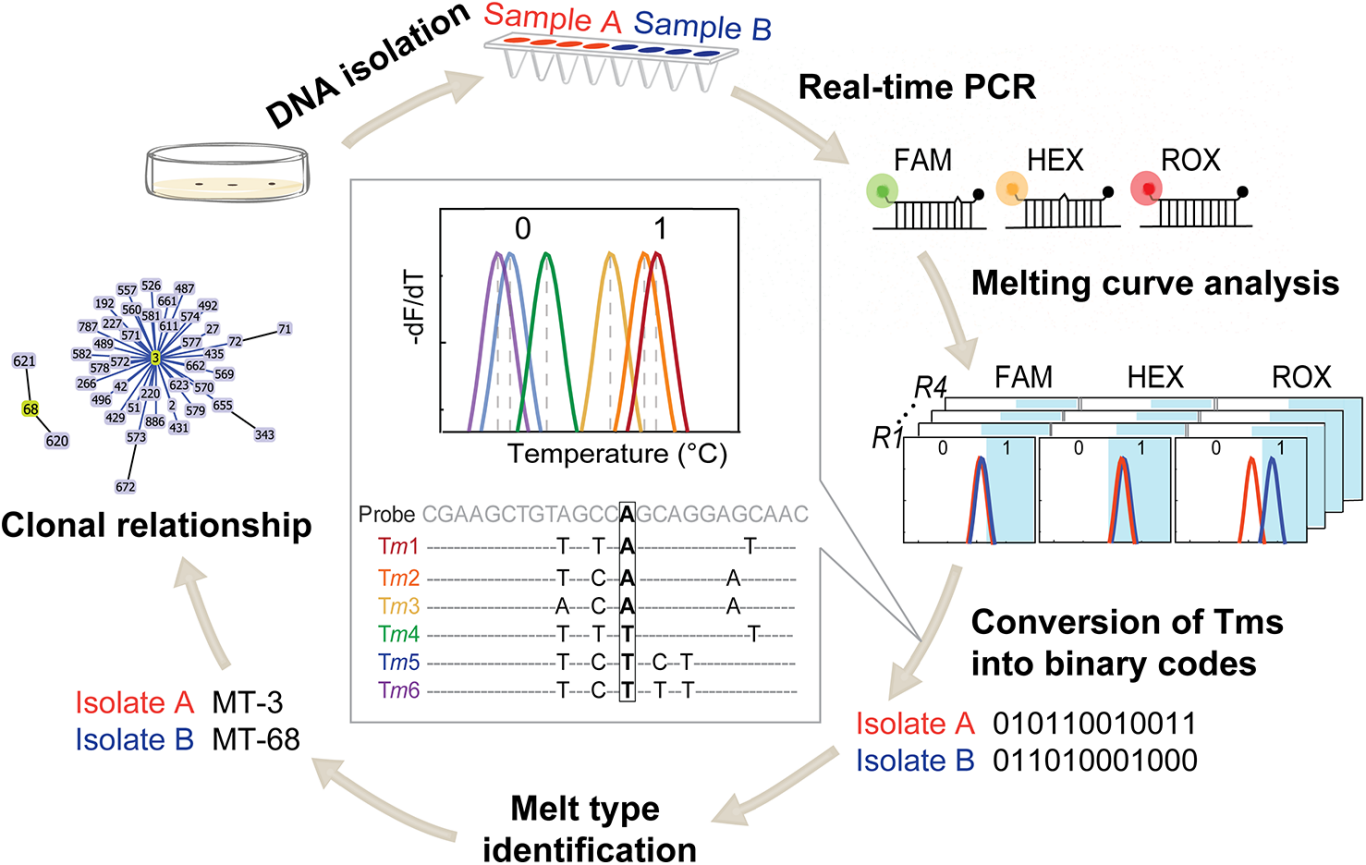
Flowchart of MLMT analysis of *V*. *parahaemolyticus*. The flowchart illustrates the typing procedure from SNP detecting to data handling. Isolated genomic DNA is first aliquoted into four PCR reactions (R1-R4). Each reaction detects three SNP sites using three differently fluorophore-labeled probes (FAM, HEX, and ROX). The produced twelve T_*m*_ values by four PCR reactions are then converted into a 12-digit binary code, which forms a melt type (MT). Isolate A (MT-3) and isolate B (MT-68) are shown as examples. The rule of converting T_*m*_s into binary codes is illustrated in the insert.

The dual-labeled, self-quenched probes alone are non-fluorescent or weakly fluorescent but become fluorescent when hybridizing with the reversely complementary single-stranded DNA. After asymmetric PCR, the produced excess single-stranded amplicons are targets for the dual-labeled, self-quenched probes. Post-PCR melting curve analysis would generate T_*m*_ values reflecting the sequence variations in the probe binding region of the amplicons. Due to the possible existence of polymorphic SNPs sites in the probe-binding regions, a series of T_*m*_ rather than a single T_*m*_ for one allelic type would be generated. The probe was designed in such a way that it is complementary with none of the sequence variants at these polymorphic SNP sites. Consequently, the T_*m*_ values for one allelic type would be always lower than another allelic type. The principle of MLMT is illustrated by diagnosing a SNP with “A” and “T” alleles (Fig 1).The probe gives allelic type “A” T_*m*_ values (T_m1_~T_m3_) higher than allelic type “T” (T_m4_~T_m6_). The lowest T_*m*_ of allelic type “A” (T_m3_) is defined as the critical T_*m*_. The allele with a T_*m*_ equal to or higher than the critical T_*m*_ is defined as binary code “1” and the allele with a T_*m*_ lower than the critical T_*m*_ is defined as “0”. A series of binary codes could be obtained when multiple SNPs are genotyped. The concatenated serial binary codes are defined as MTs. The MTs can be further linked to STs or CCs.

Extracted DNA from each isolate was analyzed in four PCR reactions. Asymmetric linear-after-the-exponential (LATE) PCR [29] was used to generate single-stranded products. Each 25-μL reaction mix contained 67 mM Tris-HCl (pH 8.8), 16 mM (NH_4_)_2_SO_4_, 0.01% (W/V) Tween 20, 3.5 mM MgCl_2_, 1 unit TaqHS DNA polymerase (Takara, Dalian, China), 200 μM of dNTPs, 0.04~0.08 μM limiting primers, 0.6~0.8 μM excess primers, 0.1~0.4 μM probes, and 5 μL of genomic DNA. The specific amount of primers and probes are given in S4 Table. The real-time PCR program was 95°C for 10 min, followed by 10 cycles of touchdown PCR (95°C for 15 s, 64°C with 1°C/cycle decrease for 15 s, and 72°C for 20 s) and 40 cycles of 95°C for 15 s, 55°C for 15 s, and 72°C for 20 s. The melting curve analysis started with a denaturing step at 95°C for 1 min, a hybridization step at 30°C for 3 min, followed by a stepwise temperature increase from 30°C to 75°C in 0.5°C increments. The fluorescence from FAM, HEX and ROX channels was recorded during the melting analysis procedure. The assay was performed on a Bio-Rad CFX 96 real-time PCR system (Bio-Rad, Hercules, CA) and the T_*m*_ values from each reaction were used for conversion into allelic types and to form the binary codes inlet.

### Concordance between MLMT and MLST

The concordance between the two typing methods was evaluated by calculating the adjusted Rand index (*AR*) [30] and the adjusted Wallace coefficient (*AW*) [31]. The *AR* was used to evaluate the overall concordance of the typing methods. *AR* takes into account that the agreement between partitions could arise by chance alone. The *Aw*es used to assess bidirectional concordance by predicting the possibility of two strains clustered together when they have been assigned into one group by another method. The values of both *AR* and *AW* range from 0 to 1, with higher values reflecting better concordance. Confidence intervals for *AR* and *AW* were estimated using a jackknife pseudo-values re-sampling method [32]. All the calculations were carried out using Comparing Partitions (http://darwin.phyloviz.net/ComparingPartitions/).

## Results

### Selection of discriminatory SNP set

A set of 12 SNPs were chosen with the cumulative SID values of 0.9974 (Table 1). The association between the ST and MT was given as a Microsoft Excel spreadsheet (S5 Table), which can be used to search for ST by MT and vice versa. The spreadsheet shows that 798 STs of *V*. *parahaemolyticus* could be resolved into 427 MTs with a SID value of 0.997 (95% CI, 0.997–0.998). Of the 427 MTs, 249 (58.3%) could be assigned each with a unique ST.

**Table 1:** The 12 informative SNPs for MLMT.

SNP No.	Name	Position	Allelic type	Binary code	Critical T_*m*_ (°C)
1	*pntA*	69	T	1	≥62.0
			C	0	<62.0
2	*tnaA*	183	T	1	≥62.5
			C	0	<62.5
3	*dtdS*	218	C	1	≥67.5
			T	0	<67.5
4	*dnaE*	382	T	1	≥52.0
			C	0	<52.0
5	*gyrB*	82	C	1	≥61.0
			T	0	<61.0
6	*dnaE*	491	T	1	≥66.5
			C	0	<66.5
7	*dtdS*	98	C	1	≥57.0
			T	0	<57.0
8	*dnaE*	518	A	1	≥58.5
			G	0	<58.5
9	*dnaE*	422	T	1	≥56.5
			C	0	<56.5
10	*gyrB*	268	T	1	≥59.5
			C	0	<59.5
11	*pyrC*	17	A	1	≥55.0
			T	0	<55.0
12	*gyrB*	304	G	1	≥61.0
			A	0	<61.0

### The critical T_*m*_s

Because the 0/1 codes for SNPs were determined by comparing T_*m*_s with the corresponding critical T_*m*_s, the reproducibility of T_*m*_ measurement was critical for the accuracy of the typing results. A collection of isolates and artificial plasmids that offer the critical T_*m*_ were tested in 10 replicates using three separately prepared batches of reaction mixes. The mean T_*m*_±SD and variation (CV %) were listed in S6 Table. The low variations in T_*m*_ measurement ensure correct interrogation of the allelic types of SNP. The critical T_*m*_s for 12 probes are listed in Table 1.

### *In silico* analysis of *V*. *parahaemolyticus* isolates

We performed an *in silico* analysis of the 1169 isolates available in the MLST database (April 15, 2014), which represented in total 589 STs (SID = 0.967, 95% CI, 0.959–0.974) in correspondence with349 MTs (SID = 0.960, 95% CI,0.952–0.969). The *AR* coefficient between MT and ST was 0.912 (95% CI, 0.875–0.946). The *AW* (MT→ST) was 0.838 (95% CI, 0.782–0.895) and the *AW* (ST→MT) was 1.000 (95% CI, 1.000–1.000). The above results indicated that MLMT had discriminatory power close to MLST.

### Evaluation of MLMT

**Fig 2:**
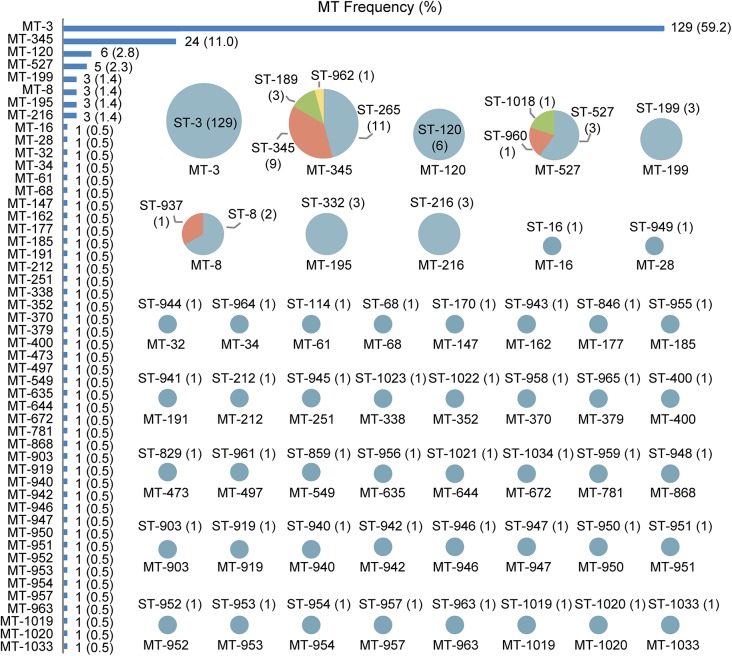
MLMT analysis results of 218 *V*. *parahaemolyticus* isolates. The frequency of each MT is given together with the number of the corresponding ST. Also given are the type and number of STs of all the MTs obtained from the 218 isolates. The size of the pies illustrates the relative number of MTs but not in a true scale.

The practical performance of MLMT was assessed using 218 *V*. *parahaemolyticus* isolates. MLMT resolved 218 isolates into 50 MTs with SID of 0.638 (95%CI, 0.563–0.712) (Fig 2 and Table 2). Among them, the most common MT was MT-3, which was composed of 129 isolates and accounted for 59.2% of the total isolates (S7 Table). The second most common MT was MT-345, which was composed of 24 isolates and accounted for 11.0% of the total isolates. Forty two MTs were each composed of one isolate.

**Table 2:** The discriminatory power of MLMT and MLST and their congruence.

Type	Number	SID (95% CI)	*AR* ^a^ (95% CI)	*AW* ^a^ (95% CI)	
			ST	MT	ST
MT	50	0.638	0.982	-	0.965
		(0.563–0.712)	(0.969–0.997)		(0.939–0.993)
ST	56	0.646	-	1.000	-
		(0.569–0.722)		(1.000–1.000)	

^a^AR: adjusted Rand index; AW: adjusted Wallace coefficient.

By comparison, MLST resolved these 218 isolates into 56 STs with SID of 0.646 (95%CI, 0.569–0.722) (Fig 2 and Table 2). Among them, ST-3 was the most prevalent sequence type comprising of 129 isolates of MT-3. Forty-six of the STs contained a single isolate, and 34 of them were newly found. The 56 STs analyzed by goeBURST produced two CCs: CC345 (ST-189, 265, 345, 962) and CC527 (ST-527, ST-960). The four STs of CC345 and two STs of CC527 corresponded to MT-345 and MT-527 respectively. The singleton ST-1018, as predicted by S5 Table, was included in MT-527. ST-937, the double-locus variant of ST-8, was clustered by MT-8 with ST-8.Association analysis between MT and ST for the 218 isolates showed that MLMT results fully agreed with the theoretical predication of the Microsoft Excel spreadsheet (S5 Table).

**Fig 3:**
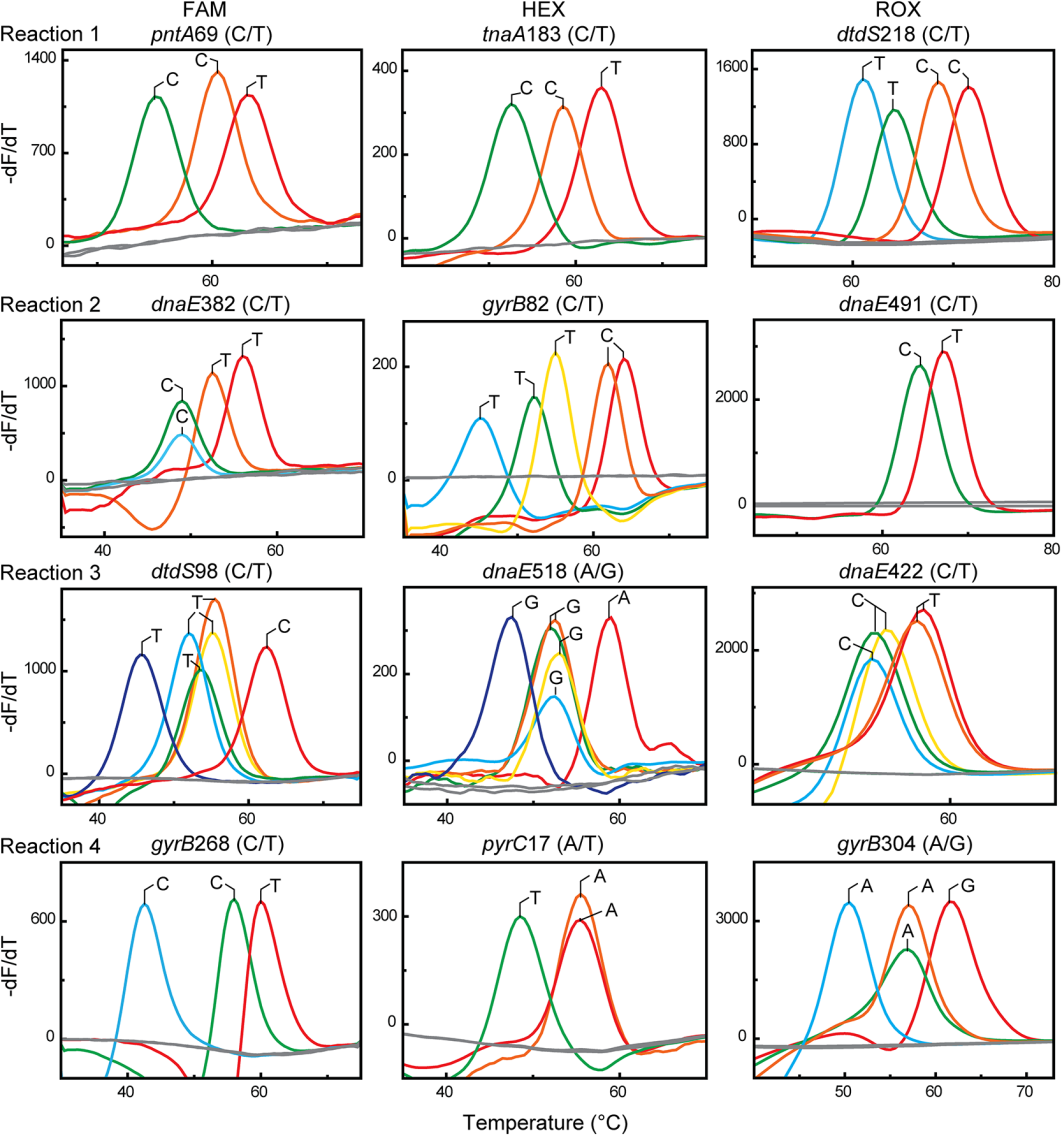
Melting curves obtained from the 218 isolates. Melting curves from those isolates displaying unique T_*m*_ values are shown in color. The non-template controls are shown in grey.

By comparing the MLMT and MLST results, we observed that the two alleles of each SNP could be completely discriminated from each other regardless of the presence of polymorphic SNPs that were encountered in these samples. The representative original melting curves presented in the 218 isolates are shown in Fig 3.

When we submitted the newly found STs to the MLST database (July, 2014), the ST number of *V*. *parahaemolyticus* had increased to 1100. We wonder whether the newly added STs might have changed the discriminatory power of the 12-SNP MLMT scheme. An updated Microsoft Excel spreadsheet was edited that includes all of the 1100 STs (S8 Table). We found that all STs could be resolved into 552 MTs with a SID of 0.998 (95% CI, 0.997–0.998). In comparison with the original database containing798 STs, the discriminatory power of MLMT remains unchanged, demonstrating the robustness of the MLMT scheme.

The congruence between MLMT and MLST was evaluated by *AR* and *AW* based on the typing results of 218 isolates (Table 2). The *AR* index between MT and ST was calculated to be 0.982 (95% CI, 0.969–0.997), showing a good overall concordance between the two typing approaches. The *AW* coefficient from MT to ST was 0.965 (95%CI, 0.960–0.971), and from ST to MT was 1.000 (95%CI, 1.000–1.000), respectively, further yielding a high congruence between the two typing methods. Altogether, the results demonstrated a high level of concordance between MT and ST in typing the 218 isolates.

**Fig 4:**
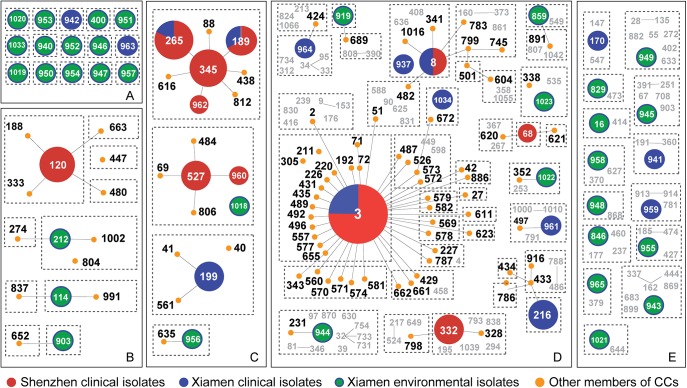
A goeBURST snapshot for population structures of 56 STs derived from 218 *V*. *parahaemolyticus* isolates superimposed by the corresponding MTs. Colored circles represent clinical isolates from Shenzhen (red), clinical isolates from Xiamen (blue), and environmental isolates from Xiamen (green). The size of the circle represents the relative abundance of the ST. The orange dots linked by grey lines represent those STs differed by a single locus variation from the ancestral ST within one CC. The boxes with dotted lines represent one MT. The numbers shown in grey color are from the MLST database but absent in this study. Panels from A to E represent five levels of relatedness between MT and ST.

For a more intuitive understanding of the performance of MLMT, the relationship between MTs and population structure of STs obtained by goeBURST was investigated for the 218 *V*.*parahaemolyticus* isolates. In order to have a comprehensive overview, we also included those STs in the database that belonged to the corresponding CCs in addition to the 56 STs found in this work. Five levels of relatedness could be classified. First, one MT corresponds with one ST (Fig 4A), demonstrating a complete concordance between MLMT and MLST. Second, one MT corresponds with subgroups of one CC (Fig 4B). For example, in CC120, MT-663, MT-447, and MT-480 correspond respectively with different STs. MT-120 corresponds with a subgroup of CC120 containing three STs, i.e., ST-120, ST-188, and ST-133.Third, one MT corresponds with one CC (Fig 4C). For example, MT-345 corresponds with CC345 that contains seven STs. Similarly, MT-199 corresponds with CC199, and MT-527 corresponds with CC527. Both CC199 and CC527 contain varied number of STs. Forth, one MT corresponds with subgroups of one CC, in which STs from other CCs might exist (Fig 4D). For example, CC3 is divided into 14 MTs, and six of which contain non-CC3 STs. Similar situation is also found in CC8 and CC332. Fifth, one MT corresponds with a group of STs from different CCs(Fig 4E). For example, although each of the 15 MTs corresponds with a single ST of the 56 STs, these MTs also contain irrelevant STs according to the prediction on 798 STs.

## Discussion

MLST is a powerful typing tool in the study of clonal relationships and population structures of bacteria. Unfortunately, its use is often hindered by cost and time when analyzing a large number of samples or when tracking epidemic spread in a timely manner. In this context, MLMT described hereby can be regarded as a simplified version of MLST with increased efficiency and cost-effectiveness. The entire MLMT procedure could be finished within 3 hours on a real-time PCR machine and the cost was approximately 50 times less than MLST. The ability of detecting multiple SNPs in one reaction further helps to simplify the operation and increase the throughput. Moreover, the interpretation of T_*m*_s into binary codes can be easily automated and the efficiency of detection can be further improved.

As a MLST derivative method, it is essential for MLMT to keep its typing results associated with MLST. Because the discriminatory SNPs of MLMT are extracted from the concatenated sequences of the housekeeping genes used by MLST, the SNPs-derived MT could thus be associated with ST. This association was given in a Microsoft Excel spreadsheet in our study (S5 Table for 798 STs and S8 Table for 1100 STs). According to the spreadsheet, every ST can be assigned to an MT, and an MT can be assigned to an ST or a group of STs. For one isolate, if the MT obtained by MLMT is included in the spreadsheet, it can be assigned to one or a group known STs in the spreadsheet though it may also represent a new ST not included in the spreadsheet. If otherwise the MT is not included in the spreadsheet, it must represent a new ST. Consequently, a known MT detected by MLMT may be or may not be a new ST, but an unknown MT detected must be a new ST. Through this association, the portable nature of MLST is kept in MLMT, making MTs accessible and exchangeable among different laboratories.

The 12-SNP MLMT scheme could discriminate the majority of STs. In order to resolve more STs, more SNPs should be included to increase the SID but at the cost of additional detection reactions. Obviously, an optimal MLMT scheme should have a high SID but with fewer SNPs. This approach however might miss some SNPs that can discriminate single locus variant (SLV) in certain CCs. For example, MT-3 contains 14 SLVs of CC3, meaning that MLMT could not discriminate all of these 14 SLVs despite its SID being as high as 0.997. To discriminate these SLVs, additional SNPs need to be searched within STs contained in MT-3. For this purpose, an association spreadsheet between MT and ST is demanded to evaluate the concordance between MTs and STs. We are now developing a software named “MTsum”, which is able to generate this spreadsheet. It can also calculate the total number of MT and the number of MTs that have a single ST. Once a set of SNPs are chosen by minimum SNPs, the generated spreadsheet can be used to guide a new round of SNPs selection for improved discrimination. After several rounds of selection, a set of SNPs with ideal discrimination can be obtained. We thus believe that by the combined use of MTsum and Minimum SNPs, an optimal SNP set that is able to discriminate the largest number of STs could be produced.

In our MLMT scheme, SNPs from the *recA* gene were excluded. Despite the high discriminatory power, *recA* is frequently recombined and is thus often not recommended as a molecular marker for evolutionary analyses of *V*. *parahaemolyticus* [8, 10, 33, 34]. Moreover, many SNPs in *recA* alleles (but not in other genes) were derived from long segment inserts of horizontal gene transfer [10, 34], and these SNPs may interfere with identification of the allelic types of the original *recA*. Therefore, the omission of *recA* could obviate the above uncertainties.

*V*. *parahaemolyticus* has a diverse population structure [35, 36]. For example, goeBURST analysis of the 798 STs in the MLST database of *V*. *parahaemolyticus* identified 94 CCs (or groups) and 513 singletons. At current stage, the 12-SNP MLMT scheme could only resolve 798 STs into 427 MTs (S5 Table). However, once the MT is known, those STs contained in it can be identified by sequencing only the discrepant loci instead of all the seven housekeeping genes. In this regards, a preliminary MLMT analysis prior to MLST could substantially save time and cost, in particular, when a large number of samples are involved.

In conclusion, we developed a melting curve-based MLST derivative, MLMT, which allows rapid and cost-effective typing of *V*. *parahaemolyticus*. The ease of use and high throughput would facilitate processing a large number of isolates. We thus expect its immediate application in clinical microbiology laboratories where real-time PCR instruments are commonly equipped.

## Supporting Information

S1 Table The sequences of probes and their three types of T_*m*_ values. (DOCX)

S2 Table MLST Primers. (DOCX)

S3 Table Real-time PCR Primers of MLMT. (DOCX)

S4 Table The amount of primers and probes used in MLMT. (DOCX)

S5 Table The conversion keys for MLMT to 798 STs (21 June, 2013) of *V*. *parahaemolyticus* MLST database. (XLSX)

S6 Table T_*m*_ reproducibility of MLMT. (DOC)

S7 Table 218 sequenced *V*.*parahaemolyticus*isolates in this study. (DOCX)

S8 Table The conversion keys for MLMT to 1100 STs (23July, 2014) of *V*. *parahaemolyticus* MLST database. (XLSX)
