# Supplementary material for: Multicolor Melting Curve Analysis-Based Multilocus Melt Typing of Vibrio parahaemolyticus
Source: PLoS One. 2015 Sep 14;10(9):e0136998. doi: 10.1371/journal.pone.0136998 (PMC4569271; doi:10.1371/journal.pone.0136998)
Supplement: S1 Table — (DOCX) [file pone.0136998.s001.docx]

| **S1 Table**. The sequences of probes and their three types of T*_m_* values. | | | | |
| --- | --- | --- | --- | --- |
|  | | *In silico*  T*_m_* (°C) | Hybridization T*_m_* (°C) | PCR  T*_m_* (°C) |
| **P1-*pntA*-69** | |  |  |  |
| FAM- TGAIATCATCAT**T+**ACAACTGCATTAATCC-BHQ1 | | | | |
| **1** | TGATATCATCAT**T**ACAACTGCATTAATCC | 61.51 | 67.0 | 63.0 |
|  | TGACATCATCAT**T**ACAACTGCATTAATCC | 61.32 | 66.5 |  |
| **0** | TGATATCATCAT**C**ACAACTGCATTAATCC | 58.31 | 64.5 | 60.5 |
|  | TGATATCATCAT**C**ACAACAGCATTAATCC | 54.53 | 60.5 |  |
|  | TGATATCATCAT**C**ACAACGGCATTAATCC | 53.44 | 59.5 | 55.0 |
|  | TGATATCATAAT**C**ACAACTGCATTAATCC | 52.41 | 58.0 |  |
|  | TGATATCATCAT**C**ACAACGGCATTAATTC | 46.24 | 55.0 |  |
| **P2-*tnaA*-183** | |  |  |  |
| HEX- GGCATGTG**T**TGTATTCGTGA-BHQ1 | | | | |
| **1** | GGCATGTG**T**TGTATTCGTGA | 60.73 | 67.0 | 63.0 |
| **0** | GGCATGTG**C**TGTATTCGTGA | 57.32 | 65.0 | 58.5 |
|  | GGCATGTG**C**TGTATTCGAGA | 51.06 | 59.5 | 52.5 |
| **P3-*dtdS*-218** | |  |  |  |
| ROX-AACTGTCGTGGIGG**C**CGTACTCAC-BHQ2 | | | | |
| **1** | AACTGTCGTGGTGG**C**CGTACTCAC | 69.93 | 74.5 | 71.5 |
|  | AACTGTCGTGGCGG**C**CGTACTCAC | 69.48 | 72.0 | 68.5 |
|  | AACTGTCGTGGAGG**C**CGTACTCAC | 67.97 | 72.0 |  |
| **0** | AACTGTCGTGGTGG**T**CGTACTCAC | 63.74 | 67.5 | 64.0 |
|  | AACTGTCGTGGCGG**T**CGTACTCAC | 62.45 | 65.0 | 61.0 |
|  | AACTGTCGTGGTGG**T**CGTACACAC | 59.14 | 62.5 |  |
| **P4-*dnaE*-382** | |  |  |  |
| FAM-TCTTCTTCIT**T+**AGGC*****AATAGGAACTCTAG-BHQ1 | | | | |
| **1** | TCTTCTTCGT**T**AGGGAATAGGAACTCTAG | 60.29 | 60.0 | 56.0 |
|  | TCTTCTTCAT**T**AGGGAATAGGAACTCTAG | 57.63 | 57.0 | 52.5 |
|  | TCTTCTTCAT**T**AGGAAATAGGAACTCTAG | 57.36 | 57.0 |  |
| **0** | TCTTCTTCTT**C**AGGGAATAGGAACTCTAG | 55.09 | 54.5 |  |
|  | TCTTCTTCAT**C**AGGGAATAGGAACTCTAG | 53.73 | 53.5 | 49.5 |
|  | TCTTCTTCAT**C**AGGAAATAGGAACTCTAG | 53.60 | 54.0 | 49.5 |
|  | TCTTCTTCCT**C**AGGGAATAGGAACTCTAG | 53.37 | 53.0 |  |
|  | TCTTCTTCAT**C**AGGGAAGAGGAACTCTAG | 49.11 | 47.5 |  |
|  | TCTTCTACAT**C**AGGGAATAGGAACTCTAG | 48.50 | 49.0 |  |
|  | TCTTCTTCAT**C**AGGGAATAAGAACTCTAG | 48.16 | 48.5 |  |
|  | TCTTCTACAT**C**TGGGAATAGGAACTCTAG | 43.21 | 49.0 |  |

(To be continued)

|  | | *In silico*  T*_m_* (°C) | | Hybridization T*_m_* (°C) | | PCR  T*_m_* (°C) |  |  |
| --- | --- | --- | --- | --- | --- | --- | --- | --- |
| **P5-*gyrB*-82** | |  | |  | |  |  |  |
| HEX-CGTGAAGACGGIAT**C**TCIGTAGAAGT-BHQ1 | |  | |  | |  |  |  |
| **1** | CGTGAAGACGGTAT**C**TCGGTAGAAGT | 64.48 | | 68.5 | | 64.0 |  |  |
|  | CGTGAAGACGGCAT**C**TCGGTAGAAGT | 63.01 | | 66.0 | | 62.0 |  |  |
|  | CGTGAAGACGGCAT**C**TCTGTAGAAGT | 62.52 | | 65.5 | |  |  |  |
| **0** | CGTGAAGACGGTAT**T**TCGGTAGAAGT | 59.76 | | 61.5 | |  |  |  |
|  | CGTGAAGACGGCAT**T**TCGGTAGAAGT | 57.83 | | 59.0 | | 55.0 |  |  |
|  | CGTGAAGACGGCAT**T**TCAGTAGAAGT | 55.00 | | 56.0 | | 52.0 |  |  |
|  | CGTGAAGACGGCAT**T**TCAGTTGAAGT | 50.84 | | 51.5 | |  |  |  |
|  | CGTGAAGATGGCAT**T**TCGGTAGAAGT | 50.13 | | 51.0 | | 45.0 |  |  |
| **P6-*dnaE*-491** | |  | |  | |  |  |  |
| ROX-AAGTATTCACC**T+**AAACGAACGGTCA-BHQ2 | | |  | |  | | |  |
| **1** | AAGTATTCACC**T**AAACGAACGGTCA | 65.51 | | 71.0 | | 67.0 |  |  |
| **0** | AAGTATTCACC**C**AAACGAACGGTCA | 62.92 | | 68.0 | | 64.5 |  |  |
|  | AAGTATTCCCC**C**AAACGAACGGTCA | 61.33 | | 66.0 | |  |  |  |
|  | AAGTATTCACC**C**AAACGTACGGTCA | 59.89 | | 63.5 | |  |  |  |
|  | AAGTATTCACC**C**AAACGAACAGTCA | 58.57 | | 63.5 | |  |  |  |
|  | AAGTATTCACC**C**AAGCGAACGGTCA | 58.58 | | 63.0 | |  |  |  |
|  | AAGTATTCACC**C**AAACGAACTGTCA | 57.10 | | 62.5 | |  |  |  |
| **P7-*dtdS*-98** | |  | |  | |  |  |  |
| FAM-TATGGTTGTGGG**C**CACGAATACGTG-BHQ1 | |  | |  | |  |  |  |
| **1** | TATGGTTGTGGG**C**CACGAGTACGTG | 67.31 | | 67.5 | |  |  |  |
|  | TATGGTTGTTGG**C**CACGAATACGTG | 65.26 | | 67.5 | | 62.5 |  |  |
|  | TATGGTAGTTGG**C**CACGAATACGTG | 62.29 | | 63.5 | |  |  |  |
| **0** | TATGGTTGTTGG**T**CACGAATACGTG | 58.26 | | 61.0 | | 55.5 |  |  |
|  | CATGGTTGTCGG**T**CACGAATACGTG | 59.17 | | 60.5 | |  |  |  |
|  | TATGGTTGTCGG**T**CACGAATACGTG | 58.15 | | 60.5 | | 55.0 |  |  |
|  | TATGGTTGTGGG**T**CACGAGTACGTG | 60.32 | | 59.5 | | 53.5 |  |  |
|  | TATGGTTGTCGG**T**CACGAATACGTA | 57.58 | | 58.0 | | 52.0 |  |  |
|  | TATGGTTATCGG**T**CACGAATACGTG | 52.90 | | 54.5 | |  |  |  |
|  | TATGGTTGTCGG**T**CACGAATATGTG | 50.68 | | 51.5 | | 45.5 |  |  |

(To be continued)

|  | | *In silico*  T*_m_* (°C) | Hybridization T*_m_* (°C) | PCR  T*_m_* (°C) |
| --- | --- | --- | --- | --- |
| **P8-dnaE-518** | | | | |
| HEX-CGTTC*****CAACGCTT**A**GCGATCTCC*****ACACT-BHQ1 | | | | |
| **1** | CGTTGCAACGCTT**A**GCGATCTCAACACT | 63.01 | 64.0 |  |
|  | CGTTACAACGCTT**A**GCGATCTCGACACT | 62.21 | 63.5 | 59.0 |
|  | CGTTACAACGCTT**A**GCGATCTCAACACT | 62.02 | 64.0 |  |
| **0** | CGTTACAACGCTT**G**GCGATCTCGACACT | 58.44 | 58.0 | 53.0 |
|  | CGTTGCAACGCTT**G**GCGATCTCGACACT | 59.47 | 57.0 | 52.5 |
|  | CGTTACAACGCTT**G**GCGATCTCAACACT | 58.35 | 58.0 | 52.5 |
|  | CGTTGCAACGCTT**G**GCGATCTCAACACT | 59.34 | 58.0 | 52.0 |
|  | CGTTACAACGCTT**G**GCGATCTCTACACT | 58.35 | 58.0 |  |
|  | CGTTACAACGCTT**G**GCAATCTCGACACT | 53.25 | 52.5 | 47.5 |
|  | CGTTACAGCGTTT**G**GCGATCTCAACACT | 44.07 | 47.0 |  |
| **P9-dnaE-422** | | | | |
| ROX-CCAAACCTTCC*****CG**T+**GACTTCATC*****ACC-BHQ2 | | | | |
| **1** | CCAAACCTTCTCG**T**GACTTCATGACC | 59.70 | 63.0 | 57.5 |
|  | CCAAACCTTCGCG**T**GACTTCATGACC | 59.06 | 62.0 |  |
|  | CCAAACCTTCTCG**T**GACTTCATAACC | 58.25 | 63.0 | 58.0 |
| **0** | CCAAACCTTCTCG**C**GACTTCATGACC | 57.10 | 59.5 | 55.0 |
|  | CCAAACCTTCACG**C**GACTTCATGACC | 56.76 | 59.5 | 54.5 |
|  | CCAAACCTTCGCG**C**GACTTCATGACC | 56.49 | 59.0 | 54.0 |
|  | CCAAACCTTCTCG**C**GACTTCATAACC | 55.77 | 60.0 |  |
|  | CCAGCCCTTCACG**C**GACTTCATGACC | 47.40 | 52.0 |  |
| **P10-gyrB-268** | | | | |
| FAM-ACGTCAGG**T**GACGATGC-BHQ1 | |  |  |  |
| **1** | ACGTCAGG**T**GACGATGC | 62.21 | 67.0 | 60.0 |
| **0** | ACGTCAGG**C**GACGATGC | 59.32 | 62.0 | 56.5 |
|  | ACATCAGG**C**GACGATGC | 52.62 | 53.5 |  |
|  | ACTTCAGG**C**GACGATGC | 51.20 | 51.0 |  |
|  | ACGTCAGG**C**GATGATGC | 49.28 | 48.0 | 42.5 |

(To be continued)

|  | | *In silico*  T*_m_* (°C) | Hybridization T*_m_* (°C) | PCR  T*_m_* (°C) |
| --- | --- | --- | --- | --- |
| **P11-*pyrC*-17** | | | | |
| HEX-CGAAGCTG*****TAG*****CC**A**GCAGGAGCAAC-BHQ1 | | | | |
| **1** | CGAAGCTTTACCC**A**GCAGGAGCAAC | 66.21 | 66.5 |  |
|  | CGAAGCTATACCC**A**GCAGGAGCAAC | 65.76 | 61.0 |  |
|  | CGAAGCTTTACCC**A**GCAGGTGCAAC | 62.04 | 61.0 | 55.5 |
|  | CGAAGCTTTATCC**A**GCAGGTGCAAC | 61.41 | 62.0 | 55.5 |
| **0** | CGAAGCTTTACCC**T**GCAGGCGCAAC | 58.49 | 57.0 |  |
|  | CGAAGCTTTACCC**T**GCAGGTGCAAC | 56.81 | 54.0 | 48.5 |
|  | CGAAGCTTTATCC**T**GCAGGTGCAAC | 55.85 | 54.5 |  |
|  | CGAAGCTTTACCC**T**GCCGGTGCAAC | 52.03 | 48.0 |  |
|  | CGAAGCTTTACCC**T**GCTGGTGCAAC | 50.66 | 46.5 |  |
|  | CGAAGCTTTACCC**T**GCGGGTGCAAC | 49.53 | 37.5 |  |
|  | CGAAGCTTTACCC**T**GCAGGTGTAAC | 47.60 | 43.0 |  |
| **P12-gyrB-304** | | | | |
| ROX-GCGGTTGTTTC**G**GTAAGAGTGCCTGA-BHQ2 | | | | |
| **1** | GCGGTTGTTTC**G**GTGAAAGTGCCTGA | 65.42 | 68.0 |  |
|  | GCCGTTGTTTC**G**GTGAGAGTGCCTGA | 63.94 | 66.5 |  |
|  | GCTGTTGTTTC**G**GTAAAAGTGCCTGA | 62.14 | 66.5 |  |
|  | GCCGTTGTTTC**G**GTAAAAGTGCCTGA | 62.08 | 66.5 | 61.5 |
| **0** | GCCGTTGTTTC**A**GTAAAAGTGCCTGA | 57.68 | 62.0 | 56.5 |
|  | GCTGTTGTTTC**A**GTAAAAGTGCCTGA | 57.62 | 62.0 | 57.0 |
|  | GCCGTTGTCTC**A**GTAAAAGTGCCTGA | 53.77 | 58.0 |  |
|  | GCCGTTGTTTC**A**GTAAAAGTGCCAGA | 53.44 | 58.0 |  |
|  | GCCGTTGTTTC**A**GTAAAAGTTCCTGA | 52.17 | 56.0 | 50.5 |

The bold letters indicate the selected SNP sites and the underlined represent the non-target variation sites. The binary codes in correspondence with the allelic types are also given.

***** Mismatched bases were introduced in probes.

**^+^** LNA modified bases
