# Supplementary material for: Multicolor Melting Curve Analysis-Based Multilocus Melt Typing of Vibrio parahaemolyticus
Source: PLoS One. 2015 Sep 14;10(9):e0136998. doi: 10.1371/journal.pone.0136998 (PMC4569271; doi:10.1371/journal.pone.0136998)
Supplement: S2 Table — (DOCX) [file pone.0136998.s002.docx]

**S2 Table.** MLST Primers

| Gene |  | Primers | Position | Length (bp) |
| --- | --- | --- | --- | --- |
| *dnaE* | *dnaE*-L-F | TCCTCATCGGCCTCATAAAGCTC | 2012 to 2832 | 821 |
|  | *dnaE*-L-R | GTTGCGATTCATGATGGTTACACAT |  |  |
| *gyrB* | *gyrB*-L-F | CTGCGTGAGCTATCGTTCTTGA | 568 to 1351 | 784 |
|  | *gyrB*-L-R | TCTTACCTTTCAGTGGTAGGATTGC |  |  |
| *recA* | *recA*-L-F | CCGCTGCGCTAGGTCAAAT | 29 to 997 | 969 |
|  | *recA*-L-R | CTGGTTGAGCTGGAGAAAGTAGCAT |  |  |
|  | *recA*-3F ^a^ | ATAACCGCGCAATGGATGTAGAAAC | 89 to 889 | 801 |
|  | *recA*-3R ^a^ | TCTTATCGCCATTGTAGCTGTACCA |  |  |
| *dtdS* | *dtdS*-1F(M13)^b^ | tgtaaaacgacggccagtTGGCCATAACGACATTCTGA | 75 to 747 | 673 |
|  | *dtdS*-L-R | GTTGAATGCCGCTGGGTTAC |  |  |
| *pntA* | *pntA*-L-F | CTGGCAGTCTTGGCGCTATC | 569 to 1286 | 718 |
|  | *pntA*-L-R | GCAGCAGGTGCTACCGATG |  |  |
| *pyrC* | *pyrC*-L-F | CGATCGAATTCTGAAAGAGCAGC | 168 to 940 | 773 |
|  | *pyrC*-L-R | CCGATTTTTCTAGCGTCACCGTAT |  |  |
| *tnaA* | *tnaA*-L-F | TCGAAGAACATCTGTGCCATC | 538 to 1192 | 655 |
|  | *tnaA*-L-R | CACGTCCGAGCAACAATGAG |  |  |

a Additional PCR and sequencing primers for recA, when isolates could not be amplified by recA-L-F/R.

b. Primer from *V. parahaemolyticus* MLST scheme (1).

**References**

1. **Gonzalez-Escalona N, Martinez-Urtaza J, Romero J, Espejo RT, Jaykus LA, DePaola A.** 2008. Determination of molecular phylogenetics of *Vibrio parahaemolyticus* strains by multilocus sequence typing. J. Bacteriol. **190:**2831-2840.
