# Supplementary material for: Multicolor Melting Curve Analysis-Based Multilocus Melt Typing of Vibrio parahaemolyticus
Source: PLoS One. 2015 Sep 14;10(9):e0136998. doi: 10.1371/journal.pone.0136998 (PMC4569271; doi:10.1371/journal.pone.0136998)
Supplement: S3 Table — (DOCX) [file pone.0136998.s003.docx]

**S3 Table**. Real-time PCR Primers

| Gene |  | | Sequence (5'-3') | Amplicon Size (bp) | |  |
| --- | --- | --- | --- | --- | --- | --- |
| *pntA* | *pntA*-F | ACTTCAATAAGAAAGCRGCAGA | | | 156 | |
|  | *pntA*-R | CCAAGTCAACAATYACRCTAC | | |  |  |
| *tnaA* | *tnaA*-F | GTGGTGATTGABTCVGCTCG | | | 248 | |
|  | *tnaA*-R | CCATAAGTGACAAAACCTTCCAT | | |  |  |
| *dtdS* | *dtdS*-F | TGCGATTGGTCAGGAAGT | | | 247 | |
|  | *dtdS*-R | GTTACCAAACGGGTCRAAGATMG | | |  |  |
| *dnaE* | *dnaE*-F | GTAAACGCTCGTCGTATTCTG | | | 210 | |
|  | *dnaE*-R | CGCTAGAAAACAGTGTYGAGAT | | |  |  |
| *gyrB* | *gyrB*-F | AAACACCAACAAAACNCCAATCATC | | | 126 | |
|  | *gyrB*-R | GTGAAACAGAAGATRTTYTCT | | |  |  |
| *dtdS* | *dtdS*-2F | GACGTACACATTTACAACTGGGACG | | | 113 | |
|  | *dtdS*-2R | CCACGAACTTCCTGRCCAAT | | |  |  |
| *gyrB* | *gyrB*-2F | CACGTACGCTAAACAGCTTTATGGA | | | 156 | |
|  | *gyrB*-2R | AAACCAGTTTATCTTTDGTTTGGCT | | |  |  |
| *pyrC* | *pyrC*-F | GGCGAACAATTCGAACCTCTGATG | | | 147 | |
|  | *pyrC*-R | GGTCACACCAGAATCTGAGT | | |  |  |
