# Supplementary material for: Multicolor Melting Curve Analysis-Based Multilocus Melt Typing of Vibrio parahaemolyticus
Source: PLoS One. 2015 Sep 14;10(9):e0136998. doi: 10.1371/journal.pone.0136998 (PMC4569271; doi:10.1371/journal.pone.0136998)
Supplement: S4 Table — (DOCX) [file pone.0136998.s004.docx]

**S4 Table**. The amount of primers and probes used in MLMT

| Reaction 1 | | Reaction 2 | | Reaction 3 | | Reaction 4 | |
| --- | --- | --- | --- | --- | --- | --- | --- |
| Primers/Probes | Amount  (pmol) | Primers/Probes | Amount  (pmol) | Primers/Probes | Amount  (pmol) | Primers/Probes | Amount  (pmol) |
| *pntA*-F | 2 | *dnaE*-F | 2 | *dtdS*-2F | 1 | *gyrB*-2F | 1 |
| *pntA*-R | 20 | *dnaE*-R | 20 | *dtdS*-2R | 20 | *gyrB*-2R | 20 |
| *tnaA*-F | 2 | *gyrB*-F | 2 | *dnaE*-F | 2 | *pyrC*-F | 1 |
| *tnaA*-R | 20 | *gyrB*-R | 20 | *dnaE*-R | 20 | *pyrC*-R | 15 |
| *dtdS*-F | 2 | *dnaE*-382-P-29 | 5 | *dtdS*-98-P-25 | 5 | *gyrB*-268-P-17 | 2.5 |
| *dtdS*-R | 20 | *gyrB*-82-P-26 | 10 | *dnaE*-518-P-28 | 5 | *pyrC*-17-P-25 | 5 |
| *pntA*-69-P-29 | 10 | *dnaE*-491-P-25 | 10 | *dnaE*-422-P-26 | 2.5 | *gyrB*-304-P-26 | 5 |
| *tnaA*-183-P-20 | 10 |  |  |  |  |  |  |
| *dtdS*-218-P-24 | 10 |  |  |  |  |  |  |
