# Supplementary material for: Multicolor Melting Curve Analysis-Based Multilocus Melt Typing of Vibrio parahaemolyticus
Source: PLoS One. 2015 Sep 14;10(9):e0136998. doi: 10.1371/journal.pone.0136998 (PMC4569271; doi:10.1371/journal.pone.0136998)
Supplement: S6 Table — (DOC) [file pone.0136998.s006.doc]

**S5**.**Table** T*_m_* reproducibility of MLMT

| Reaction | Probe | Mean T*_m_*±SD^a^(°C) | CV(%) | Intra-assay  (%, CV) | Inter-assay^b^  (%, CV) |
| --- | --- | --- | --- | --- | --- |
| **1** | *pntA*-69-P-29 | 60.70±0.25 | 0.41 | 0.31 | 0.34 |
|  | *tnaA*-183-P-20 | 63.05±0.25 | 0.39 | 0.20 | 0.39 |
|  | *dtdS*-218-P-24 | 60.95±0.31 | 0.52 | 0.29 | 0.59 |
| **2** | *dnaE*-382-P-29 | 52.79±0.25 | 0.48 | 0.32 | 0.43 |
|  | *gyrB*-82-P-26 | 64.18±0.41 | 0.64 | 0.22 | 0.30 |
|  | *dnaE*-491-P-25 | 64.70±0.42 | 0.64 | 0.08 | 0.14 |
| **3** | *dtdS*-98-P-25 | 55.04±0.23 | 0.42 | 0.19 | 0.46 |
|  | *dnaE*-518-P-28 | 58.91±0.20 | 0.33 | 0.15 | 0.29 |
|  | *dnaE*-422-P-26 | 54.70±0.25 | 0.45 | 0.16 | 0.35 |
| **4** | *gyrB*-268-P-17 | 56.05±0.16 | 0.28 | 0.09 | 0.16 |
|  | *pyrC*-17-P-25 | 55.54±0.19 | 0.34 | 0.15 | 0.28 |
|  | *gyrB*-304-P-26 | 61.55±0.16 | 0.26 | 0.11 | 0.41 |

1. Reproducibility for 28 isolates of ST-3.

b. One isolate of ST-3 at concentration of 10^3^copies/μL was tested in 10 replicates in three individually prepared batches of reaction mixes.
