# Supplementary material for: Multicolor Melting Curve Analysis-Based Multilocus Melt Typing of Vibrio parahaemolyticus
Source: PLoS One. 2015 Sep 14;10(9):e0136998. doi: 10.1371/journal.pone.0136998 (PMC4569271; doi:10.1371/journal.pone.0136998)
Supplement: S7 Table — (DOCX) [file pone.0136998.s007.docx]

**S6 Table**. 218 sequenced *V.parahaemolyticus*isolates in this study

| **Year** | **Region^a^** | **Source** | **Isolate** | **Tm Profiles (°C)** | **MT Profiles** | **MT** | **ST^b^** | **Allele profiles^c^** |
| --- | --- | --- | --- | --- | --- | --- | --- | --- |
| **2007** | SZ | clinical | 07088 | 60.5 63.0 61.0 53.0 64.5 65.0 55.0 59.0 55.0 56.0 55.5 61.5 | 010110010011 | 3 | 3 | 3, 4, 19, 4, 29, 4, 22 |
| **2007** | SZ | clinical | 07142 | 60.5 63.0 61.0 53.0 64.5 65.0 55.0 59.0 55.0 56.0 55.5 61.5 | 010110010011 | 3 | 3 | 3, 4, 19, 4, 29, 4, 22 |
| **2007** | SZ | clinical | 07165 | 60.5 63.0 61.0 53.0 64.5 65.0 55.0 59.0 55.0 56.0 55.5 61.5 | 010110010011 | 3 | 3 | 3, 4, 19, 4, 29, 4, 22 |
| **2007** | SZ | clinical | 07195 | 60.5 63.0 61.0 53.0 64.5 65.0 55.0 59.0 55.0 56.0 55.5 61.5 | 010110010011 | 3 | 3 | 3, 4, 19, 4, 29, 4, 22 |
| **2007** | SZ | clinical | 07075 | 60.5 63.0 61.0 53.5 64.5 65.0 55.0 59.0 55.0 56.0 55.5 61.5 | 010110010011 | 3 | 3 | 3, 4, 19, 4, 29, 4, 22 |
| **2007** | SZ | clinical | 07087 | 61.0 63.5 61.5 53.0 64.5 65.0 55.5 59.0 55.5 56.5 56.0 62.0 | 010110010011 | 3 | 3 | 3, 4, 19, 4, 29, 4, 22 |
| **2007** | SZ | clinical | 07092 | 60.5 63.0 61.0 53.0 64.0 65.0 55.0 59.0 55.0 56.0 55.5 61.5 | 010110010011 | 3 | 3 | 3, 4, 19, 4, 29, 4, 22 |
| **2007** | SZ | clinical | 07095 | 60.5 63.5 61.0 52.5 64.5 65.0 55.0 59.5 55.0 56.0 55.5 61.5 | 010110010011 | 3 | 3 | 3, 4, 19, 4, 29, 4, 22 |
| **2007** | SZ | clinical | 07099 | 60.5 63.0 61.0 53.0 64.0 65.0 55.5 59.0 55.0 56.0 55.5 61.5 | 010110010011 | 3 | 3 | 3, 4, 19, 4, 29, 4, 22 |
| **2007** | SZ | clinical | 07149 | 60.5 63.0 61.0 53.0 64.5 65.5 55.0 59.0 55.0 56.0 55.5 61.5 | 010110010011 | 3 | 3 | 3, 4, 19, 4, 29, 4, 22 |
| **2007** | SZ | clinical | 07256 | 60.5 63.0 61.5 53.0 64.5 65.0 55.0 59.0 55.0 56.0 55.5 61.5 | 010110010011 | 3 | 3 | 3, 4, 19, 4, 29, 4, 22 |
| **2007** | SZ | clinical | 07267 | 60.5 63.0 61.0 53.5 64.5 65.0 55.0 59.0 55.0 56.0 55.5 61.5 | 010110010011 | 3 | 3 | 3, 4, 19, 4, 29, 4, 22 |
| **2007** | SZ | clinical | 07269 | 61.0 63.5 61.5 53.0 64.5 65.0 55.5 59.5 55.0 56.5 56.0 62.0 | 010110010011 | 3 | 3 | 3, 4, 19, 4, 29, 4, 22 |
| **2007** | SZ | clinical | 07270 | 61.0 63.5 61.5 53.0 64.0 65.0 55.5 59.0 55.0 56.5 56.0 62.0 | 010110010011 | 3 | 3 | 3, 4, 19, 4, 29, 4, 22 |
| **2007** | SZ | clinical | 07271 | 60.5 63.0 61.0 53.0 64.5 65.0 55.0 59.0 55.5 56.0 55.5 61.5 | 010110010011 | 3 | 3 | 3, 4, 19, 4, 29, 4, 22 |
| **2007** | SZ | clinical | 07272 | 60.5 63.0 61.0 53.0 64.5 65.0 55.0 59.0 55.0 56.0 55.5 61.5 | 010110010011 | 3 | 3 | 3, 4, 19, 4, 29, 4, 22 |
| **2008** | SZ | clinical | 08118 | 61.0 63.5 61.0 52.5 64.0 65.0 55.5 59.0 55.0 56.0 55.5 61.5 | 010110010011 | 3 | 3 | 3, 4, 19, 4, 29, 4, 22 |
| **2008** | SZ | clinical | 08174 | 61.0 63.0 61.0 53.0 64.5 65.0 55.0 59.0 55.0 56.0 55.5 61.5 | 010110010011 | 3 | 3 | 3, 4, 19, 4, 29, 4, 22 |
| **2008** | SZ | clinical | 08125 | 61.0 63.0 61.0 53.0 64.5 65.0 55.0 59.5 54.5 56.0 55.5 61.5 | 010110010011 | 3 | 3 | 3, 4, 19, 4, 29, 4, 22 |
| **2008** | SZ | clinical | 08202 | 61.0 63.5 60.5 53.0 64.5 65.0 55.0 59.0 54.5 56.0 55.5 61.0 | 010110010011 | 3 | 3 | 3, 4, 19, 4, 29, 4, 22 |
| **2008** | SZ | clinical | 08240 | 61.0 63.0 61.0 53.0 64.5 65.0 55.0 59.0 54.5 56.0 55.5 61.5 | 010110010011 | 3 | 3 | 3, 4, 19, 4, 29, 4, 22 |
| **2011** | SZ | clinical | 11008 | 61.0 63.5 61.5 53.0 64.5 65.0 55.0 59.0 55.0 56.0 55.5 61.5 | 010110010011 | 3 | 3 | 3, 4, 19, 4, 29, 4, 22 |
| **2011** | SZ | clinical | 11015 | 60.5 63.0 61.0 53.0 64.5 65.0 55.0 59.0 55.0 56.0 55.5 61.5 | 010110010011 | 3 | 3 | 3, 4, 19, 4, 29, 4, 22 |
| **2011** | SZ | clinical | 11118 | 60.5 63.0 60.5 53.0 64.5 65.0 55.0 59.0 54.5 56.0 55.5 61.5 | 010110010011 | 3 | 3 | 3, 4, 19, 4, 29, 4, 22 |
| **2011** | SZ | clinical | 11138 | 61.0 63.0 61.0 53.0 64.5 65.0 55.0 59.0 54.5 56.0 55.5 61.5 | 010110010011 | 3 | 3 | 3, 4, 19, 4, 29, 4, 22 |
| **2011** | SZ | clinical | 11176 | 61.0 63.0 61.0 53.0 64.5 65.0 55.0 59.0 54.5 56.0 55.5 61.5 | 010110010011 | 3 | 3 | 3, 4, 19, 4, 29, 4, 22 |
| **2011** | SZ | clinical | 11180 | 61.0 63.0 61.0 53.0 64.5 65.0 55.0 59.0 54.5 56.0 55.5 61.5 | 010110010011 | 3 | 3 | 3, 4, 19, 4, 29, 4, 22 |
| **2011** | SZ | clinical | 11193 | 61.0 63.0 61.0 53.0 64.5 65.0 55.0 59.0 54.5 56.0 55.5 61.5 | 010110010011 | 3 | 3 | 3, 4, 19, 4, 29, 4, 22 |
| **2011** | SZ | clinical | 11216 | 61.0 63.0 61.0 53.0 64.5 65.0 55.0 59.0 54.5 56.0 55.5 61.5 | 010110010011 | 3 | 3 | 3, 4, 19, 4, 29, 4, 22 |
| **2011** | SZ | clinical | 11020 | 61.0 63.0 61.0 53.0 64.5 65.0 55.0 59.0 54.5 56.0 55.5 61.5 | 010110010011 | 3 | 3 | 3, 4, 19, 4, 29, 4, 22 |
| **2011** | SZ | clinical | 11030 | 61.0 63.5 60.5 53.0 64.5 65.0 55.0 59.0 54.5 56.0 55.5 61.0 | 010110010011 | 3 | 3 | 3, 4, 19, 4, 29, 4, 22 |
| **2011** | SZ | clinical | 11034 | 61.0 63.5 60.5 53.0 64.5 65.0 55.0 59.0 54.5 56.0 55.5 61.0 | 010110010011 | 3 | 3 | 3, 4, 19, 4, 29, 4, 22 |
| **2011** | SZ | clinical | 11041 | 61.0 63.5 60.5 53.0 64.5 65.0 55.0 59.0 54.5 56.0 55.5 61.0 | 010110010011 | 3 | 3 | 3, 4, 19, 4, 29, 4, 22 |
| **2011** | SZ | clinical | 11045 | 61.0 63.5 60.5 53.0 64.5 65.0 55.0 59.0 54.5 56.0 55.5 61.0 | 010110010011 | 3 | 3 | 3, 4, 19, 4, 29, 4, 22 |
| **2011** | SZ | clinical | 11046 | 61.0 63.5 60.5 53.0 64.5 65.0 55.0 59.0 54.5 56.0 55.5 61.0 | 010110010011 | 3 | 3 | 3, 4, 19, 4, 29, 4, 22 |
| **2011** | SZ | clinical | 11050 | 61.0 63.5 60.5 53.0 64.5 65.0 55.0 59.0 54.5 56.0 55.5 61.0 | 010110010011 | 3 | 3 | 3, 4, 19, 4, 29, 4, 22 |
| **2011** | SZ | clinical | 11075 | 61.0 63.5 60.5 53.0 64.5 65.0 55.0 59.0 54.5 56.0 55.5 61.0 | 010110010011 | 3 | 3 | 3, 4, 19, 4, 29, 4, 22 |
| **2011** | SZ | clinical | 11085 | 61.0 63.5 60.5 53.0 64.5 65.0 55.0 59.0 54.5 56.0 55.5 61.0 | 010110010011 | 3 | 3 | 3, 4, 19, 4, 29, 4, 22 |
| **2011** | SZ | clinical | 11087 | 61.0 63.5 60.5 53.0 64.5 65.0 55.0 59.0 54.5 56.0 55.5 61.0 | 010110010011 | 3 | 3 | 3, 4, 19, 4, 29, 4, 22 |
| **2011** | SZ | clinical | 11106 | 61.0 63.5 60.5 53.0 64.5 65.0 55.0 59.0 54.5 56.0 55.5 61.0 | 010110010011 | 3 | 3 | 3, 4, 19, 4, 29, 4, 22 |
| **2011** | SZ | clinical | 11108 | 61.0 63.5 60.5 53.0 64.5 65.0 55.0 59.0 54.5 56.0 55.5 61.0 | 010110010011 | 3 | 3 | 3, 4, 19, 4, 29, 4, 22 |
| **2011** | SZ | clinical | 11112 | 61.0 63.5 60.5 53.0 64.5 65.0 55.0 59.0 54.5 56.0 55.5 61.0 | 010110010011 | 3 | 3 | 3, 4, 19, 4, 29, 4, 22 |
| **2011** | SZ | clinical | 11113 | 61.0 63.5 60.5 53.0 64.5 65.0 55.0 59.0 54.5 56.0 55.5 61.0 | 010110010011 | 3 | 3 | 3, 4, 19, 4, 29, 4, 22 |
| **2011** | SZ | clinical | 11114 | 61.0 63.5 60.5 53.0 64.5 65.0 55.0 59.0 54.5 56.0 55.5 61.0 | 010110010011 | 3 | 3 | 3, 4, 19, 4, 29, 4, 22 |
| **2011** | SZ | clinical | 11120 | 61.0 63.5 60.5 53.0 64.5 65.0 55.0 59.0 54.5 56.0 55.5 61.0 | 010110010011 | 3 | 3 | 3, 4, 19, 4, 29, 4, 22 |
| **2011** | SZ | clinical | 11122 | 61.0 63.5 60.5 53.0 64.5 65.0 55.0 59.0 54.5 56.0 55.5 61.0 | 010110010011 | 3 | 3 | 3, 4, 19, 4, 29, 4, 22 |
| **2011** | SZ | clinical | 11123 | 61.0 63.5 60.5 53.0 64.5 65.0 55.0 59.0 54.5 56.0 55.5 61.0 | 010110010011 | 3 | 3 | 3, 4, 19, 4, 29, 4, 22 |
| **2011** | SZ | clinical | 11124 | 61.0 63.5 60.5 53.0 64.5 65.0 55.0 59.0 54.5 56.0 55.5 61.0 | 010110010011 | 3 | 3 | 3, 4, 19, 4, 29, 4, 22 |
| **2011** | SZ | clinical | 11127 | 61.0 63.5 60.5 53.0 64.5 65.0 55.0 59.0 54.5 56.0 55.5 61.0 | 010110010011 | 3 | 3 | 3, 4, 19, 4, 29, 4, 22 |
| **2011** | SZ | clinical | 11128 | 61.0 63.5 60.5 53.0 64.5 65.0 55.0 59.0 54.5 56.0 55.5 61.0 | 010110010011 | 3 | 3 | 3, 4, 19, 4, 29, 4, 22 |
| **2011** | SZ | clinical | 11131 | 61.0 63.5 60.5 53.0 64.5 65.0 55.0 59.0 54.5 56.0 55.5 61.0 | 010110010011 | 3 | 3 | 3, 4, 19, 4, 29, 4, 22 |
| **2011** | SZ | clinical | 11133 | 61.0 63.5 60.5 53.0 64.5 65.0 55.0 59.0 54.5 56.0 55.5 61.0 | 010110010011 | 3 | 3 | 3, 4, 19, 4, 29, 4, 22 |
| **2011** | SZ | clinical | 11135 | 61.0 63.5 60.5 53.0 64.5 65.0 55.0 59.0 54.5 56.0 55.5 61.0 | 010110010011 | 3 | 3 | 3, 4, 19, 4, 29, 4, 22 |
| **2011** | SZ | clinical | 11136 | 61.0 63.5 60.5 53.0 64.5 65.0 55.0 59.0 54.5 56.0 55.5 61.0 | 010110010011 | 3 | 3 | 3, 4, 19, 4, 29, 4, 22 |
| **2011** | SZ | clinical | 11140 | 61.0 63.5 60.5 53.0 64.5 65.0 55.0 59.0 54.5 56.0 55.5 61.0 | 010110010011 | 3 | 3 | 3, 4, 19, 4, 29, 4, 22 |
| **2011** | SZ | clinical | 11142 | 61.0 63.5 60.5 53.0 64.5 65.0 55.0 59.0 54.5 56.0 55.5 61.0 | 010110010011 | 3 | 3 | 3, 4, 19, 4, 29, 4, 22 |
| **2011** | SZ | clinical | 11143 | 61.0 63.5 60.5 53.0 64.5 65.0 55.0 59.0 54.5 56.0 55.5 61.0 | 010110010011 | 3 | 3 | 3, 4, 19, 4, 29, 4, 22 |
| **2011** | SZ | clinical | 11146 | 61.0 63.5 60.5 53.0 64.5 65.0 55.0 59.0 54.5 56.0 55.5 61.0 | 010110010011 | 3 | 3 | 3, 4, 19, 4, 29, 4, 22 |
| **2011** | SZ | clinical | 11150 | 61.0 63.5 60.5 53.0 64.5 65.0 55.0 59.0 54.5 56.0 55.5 61.0 | 010110010011 | 3 | 3 | 3, 4, 19, 4, 29, 4, 22 |
| **2011** | SZ | clinical | 11153 | 61.0 63.5 60.5 53.0 64.5 65.0 55.0 59.0 54.5 56.0 55.5 61.0 | 010110010011 | 3 | 3 | 3, 4, 19, 4, 29, 4, 22 |
| **2011** | SZ | clinical | 11154 | 61.0 63.5 60.5 53.0 64.5 65.0 55.0 59.0 54.5 56.0 55.5 61.0 | 010110010011 | 3 | 3 | 3, 4, 19, 4, 29, 4, 22 |
| **2011** | SZ | clinical | 11155 | 61.0 63.5 60.5 53.0 64.5 65.0 55.0 59.0 54.5 56.0 55.5 61.0 | 010110010011 | 3 | 3 | 3, 4, 19, 4, 29, 4, 22 |
| **2011** | SZ | clinical | 11157 | 61.0 63.5 60.5 53.0 64.5 65.0 55.0 59.0 54.5 56.0 55.5 61.0 | 010110010011 | 3 | 3 | 3, 4, 19, 4, 29, 4, 22 |
| **2011** | SZ | clinical | 11172 | 61.0 63.5 60.5 53.0 64.5 65.0 55.0 59.0 54.5 56.0 55.5 61.0 | 010110010011 | 3 | 3 | 3, 4, 19, 4, 29, 4, 22 |
| **2011** | SZ | clinical | 11173 | 61.0 63.5 60.5 53.0 64.5 65.0 55.0 59.0 54.5 56.0 55.5 61.0 | 010110010011 | 3 | 3 | 3, 4, 19, 4, 29, 4, 22 |
| **2011** | SZ | clinical | 11174 | 61.0 63.5 60.5 53.0 64.5 65.0 55.0 59.0 54.5 56.0 55.5 61.0 | 010110010011 | 3 | 3 | 3, 4, 19, 4, 29, 4, 22 |
| **2011** | SZ | clinical | 11177 | 61.0 63.5 60.5 53.0 64.5 65.0 55.0 59.0 54.5 56.0 55.5 61.0 | 010110010011 | 3 | 3 | 3, 4, 19, 4, 29, 4, 22 |
| **2011** | SZ | clinical | 11179 | 61.0 63.5 60.5 53.0 64.5 65.0 55.0 59.0 54.5 56.0 55.5 61.0 | 010110010011 | 3 | 3 | 3, 4, 19, 4, 29, 4, 22 |
| **2011** | SZ | clinical | 11182 | 61.0 63.5 60.5 53.0 64.5 65.0 55.0 59.0 54.5 56.0 55.5 61.0 | 010110010011 | 3 | 3 | 3, 4, 19, 4, 29, 4, 22 |
| **2011** | SZ | clinical | 11184 | 61.0 63.5 60.5 53.0 64.5 65.0 55.0 59.0 54.5 56.0 55.5 61.0 | 010110010011 | 3 | 3 | 3, 4, 19, 4, 29, 4, 22 |
| **2011** | SZ | clinical | 11186 | 61.0 63.5 60.5 53.0 64.5 65.0 55.0 59.0 54.5 56.0 55.5 61.0 | 010110010011 | 3 | 3 | 3, 4, 19, 4, 29, 4, 22 |
| **2011** | SZ | clinical | 11188 | 61.0 63.5 60.5 53.0 64.5 65.0 55.0 59.0 54.5 56.0 55.5 61.0 | 010110010011 | 3 | 3 | 3, 4, 19, 4, 29, 4, 22 |
| **2011** | SZ | clinical | 11191 | 61.0 63.5 60.5 53.0 64.5 65.0 55.0 59.0 54.5 56.0 55.5 61.0 | 010110010011 | 3 | 3 | 3, 4, 19, 4, 29, 4, 22 |
| **2011** | SZ | clinical | 11192 | 61.0 63.5 60.5 53.0 64.5 65.0 55.0 59.0 54.5 56.0 55.5 61.0 | 010110010011 | 3 | 3 | 3, 4, 19, 4, 29, 4, 22 |
| **2011** | SZ | clinical | 11194 | 61.0 63.5 60.5 53.0 64.5 65.0 55.0 59.0 54.5 56.0 55.5 61.0 | 010110010011 | 3 | 3 | 3, 4, 19, 4, 29, 4, 22 |
| **2011** | SZ | clinical | 11195 | 61.0 63.5 60.5 53.0 64.5 65.0 55.0 59.0 54.5 56.0 55.5 61.0 | 010110010011 | 3 | 3 | 3, 4, 19, 4, 29, 4, 22 |
| **2011** | SZ | clinical | 11214 | 61.0 63.5 60.5 53.0 64.5 65.0 55.0 59.0 54.5 56.0 55.5 61.0 | 010110010011 | 3 | 3 | 3, 4, 19, 4, 29, 4, 22 |
| **2011** | SZ | clinical | 11217 | 61.0 63.5 60.5 53.0 64.5 65.0 55.0 59.0 54.5 56.0 55.5 61.0 | 010110010011 | 3 | 3 | 3, 4, 19, 4, 29, 4, 22 |
| **2011** | SZ | clinical | 11218 | 61.0 63.5 60.5 53.0 64.5 65.0 55.0 59.0 54.5 56.0 55.5 61.0 | 010110010011 | 3 | 3 | 3, 4, 19, 4, 29, 4, 22 |
| **2011** | SZ | clinical | 11219 | 61.0 63.5 60.5 53.0 64.5 65.0 55.0 59.0 54.5 56.0 55.5 61.0 | 010110010011 | 3 | 3 | 3, 4, 19, 4, 29, 4, 22 |
| **2011** | SZ | clinical | 11221 | 61.0 63.5 60.5 53.0 64.5 65.0 55.0 59.0 54.5 56.0 55.5 61.0 | 010110010011 | 3 | 3 | 3, 4, 19, 4, 29, 4, 22 |
| **2011** | SZ | clinical | 11222 | 61.0 63.5 60.5 53.0 64.5 65.0 55.0 59.0 54.5 56.0 55.5 61.0 | 010110010011 | 3 | 3 | 3, 4, 19, 4, 29, 4, 22 |
| **2011** | SZ | clinical | 11223 | 61.0 63.5 60.5 53.0 64.5 65.0 55.0 59.0 54.5 56.0 55.5 61.0 | 010110010011 | 3 | 3 | 3, 4, 19, 4, 29, 4, 22 |
| **2011** | SZ | clinical | 11224 | 61.0 63.5 60.5 53.0 64.5 65.0 55.0 59.0 54.5 56.0 55.5 61.0 | 010110010011 | 3 | 3 | 3, 4, 19, 4, 29, 4, 22 |
| **2011** | SZ | clinical | 11225 | 61.0 63.5 60.5 53.0 64.5 65.0 55.0 59.0 54.5 56.0 55.5 61.0 | 010110010011 | 3 | 3 | 3, 4, 19, 4, 29, 4, 22 |
| **2011** | SZ | clinical | 11226 | 61.0 63.5 60.5 53.0 64.5 65.0 55.0 59.0 54.5 56.0 55.5 61.0 | 010110010011 | 3 | 3 | 3, 4, 19, 4, 29, 4, 22 |
| **2011** | SZ | clinical | 11227 | 61.0 63.5 60.5 53.0 64.5 65.0 55.0 59.0 54.5 56.0 55.5 61.0 | 010110010011 | 3 | 3 | 3, 4, 19, 4, 29, 4, 22 |
| **2011** | SZ | clinical | 11229 | 61.0 63.5 60.5 53.0 64.5 65.0 55.0 59.0 54.5 56.0 55.5 61.0 | 010110010011 | 3 | 3 | 3, 4, 19, 4, 29, 4, 22 |
| **2011** | SZ | clinical | 11230 | 61.0 63.5 60.5 53.0 64.5 65.0 55.0 59.0 54.5 56.0 55.5 61.0 | 010110010011 | 3 | 3 | 3, 4, 19, 4, 29, 4, 22 |
| **2011** | SZ | clinical | 11231 | 61.0 63.5 60.5 53.0 64.5 65.0 55.0 59.0 54.5 56.0 55.5 61.0 | 010110010011 | 3 | 3 | 3, 4, 19, 4, 29, 4, 22 |
| **2011** | SZ | clinical | 11232 | 61.0 63.5 60.5 53.0 64.5 65.0 55.0 59.0 54.5 56.0 55.5 61.0 | 010110010011 | 3 | 3 | 3, 4, 19, 4, 29, 4, 22 |
| **2011** | SZ | clinical | 11233 | 61.0 63.5 60.5 53.0 64.5 65.0 55.0 59.0 54.5 56.0 55.5 61.0 | 010110010011 | 3 | 3 | 3, 4, 19, 4, 29, 4, 22 |
| **2011** | SZ | clinical | 11234 | 61.0 63.5 60.5 53.0 64.5 65.0 55.0 59.0 54.5 56.0 55.5 61.0 | 010110010011 | 3 | 3 | 3, 4, 19, 4, 29, 4, 22 |
| **2011** | SZ | clinical | 11235 | 61.0 63.5 60.5 53.0 64.5 65.0 55.0 59.0 54.5 56.0 55.5 61.0 | 010110010011 | 3 | 3 | 3, 4, 19, 4, 29, 4, 22 |
| **2011** | SZ | clinical | 11236 | 61.0 63.5 60.5 53.0 64.5 65.0 55.0 59.0 54.5 56.0 55.5 61.0 | 010110010011 | 3 | 3 | 3, 4, 19, 4, 29, 4, 22 |
| **2011** | SZ | clinical | 11238 | 61.0 63.5 60.5 53.0 64.5 65.0 55.0 59.0 54.5 56.0 55.5 61.0 | 010110010011 | 3 | 3 | 3, 4, 19, 4, 29, 4, 22 |
| **2011** | SZ | clinical | 11241 | 61.0 63.5 60.5 53.0 64.5 65.0 55.0 59.0 54.5 56.0 55.5 61.0 | 010110010011 | 3 | 3 | 3, 4, 19, 4, 29, 4, 22 |
| **2011** | SZ | clinical | 11132 | 60.5 63.0 68.5 50.0 64.5 67.5 45.5 52.0 54.0 56.0 55.5 61.5 | 011011000011 | 8 | 8 | 28, 4, 82, 88, 63, 69, 1 |
| **2011** | SZ | clinical | 11220 | 61.0 63.0 68.5 50.0 62.5 65.0 55.5 47.5 58.0 56.0 48.5 57.0 | 011010001000 | 68 | 68 | 41, 40, 36, 41, 36, 39, 32 |
| **2008** | SZ | clinical | 08328 | 63.5 63.0 61.5 50.0 62.5 65.0 55.0 53.0 54.5 56.5 48.5 50.0 | 110010000000 | 120 | 120 | 60, 108, 86, 98, 18, 45, 51 |
| **2011** | SZ | clinical | 11003 | 64.0 63.5 61.5 50.0 62.5 65.0 55.0 53.0 54.5 56.5 48.5 50.0 | 110010000000 | 120 | 120 | 60, 108, 86, 98, 18, 45, 51 |
| **2011** | SZ | clinical | 11004 | 64.0 63.5 61.5 50.0 62.5 65.0 55.0 53.0 54.5 56.5 48.5 50.0 | 110010000000 | 120 | 120 | 60, 108, 86, 98, 18, 45, 51 |
| **2011** | SZ | clinical | 11052 | 64.0 63.5 61.5 50.0 62.5 65.0 55.0 52.0 54.0 56.5 48.5 50.0 | 110010000000 | 120 | 120 | 60, 108, 86, 98, 18, 45, 51 |
| **2011** | SZ | clinical | 11095 | 63.5 63.0 61.0 49.5 62.0 65.0 55.0 52.5 54.0 56.5 48.5 50.0 | 110010000000 | 120 | 120 | 60, 108, 86, 98, 18, 45, 51 |
| **2011** | SZ | clinical | 11134 | 64.0 63.5 61.5 50.0 62.5 65.0 55.0 52.5 54.0 56.5 48.5 50.0 | 110010000000 | 120 | 120 | 60, 108, 86, 98, 18, 45, 51 |
| **2011** | SZ | clinical | 11001 | 60.5 63.0 68.5 50.5 52.5 65.0 62.5 53.0 54.0 56.5 48.5 56.5 | 011000100000 | 195 | 332 | 14, 30, 141, 78, 4, 37, 13 |
| **2011** | SZ | clinical | 11073 | 60.5 63.0 68.5 50.5 52.5 65.0 62.5 52.5 54.0 56.5 48.5 56.5 | 011000100000 | 195 | 332 | 14, 30, 141, 78, 4, 37, 13 |
| **2011** | SZ | clinical | 11121 | 60.5 63.0 68.5 50.5 52.5 65.0 62.5 52.5 54.0 56.5 48.5 57.0 | 011000100000 | 195 | 332 | 14, 30, 141, 78, 4, 37, 13 |
| **2007** | SZ | clinical | 07001 | 63.5 62.5 61.0 53.0 62.5 65.0 55.0 53.0 55.0 57.0 48.5 57.0 | 110110000000 | 345 | 265 | 11, 48, 107, 48, 26, 48, 26 |
| **2007** | SZ | clinical | 07230 | 63.5 63.0 61.0 53.0 62.5 65.0 55.0 53.0 55.0 57.0 48.5 57.0 | 110110000000 | 345 | 345 | 11, 48, 19, 48, 26, 48, 26 |
| **2007** | SZ | clinical | 07002-1 | 63.5 63.0 61.0 53.0 62.5 65.0 55.0 53.0 55.0 56.5 48.5 57.0 | 110110000000 | 345 | 345 | 11, 48, 19, 48, 26, 48, 26 |
| **2007** | SZ | clinical | 07002-2 | 63.5 63.0 61.0 53.0 62.5 65.0 55.0 53.0 54.5 56.5 48.5 57.0 | 110110000000 | 345 | 345 | 11, 48, 19, 48, 26, 48, 26 |
| **2007** | SZ | clinical | 07278-1 | 63.5 63.0 61.0 53.0 62.5 65.0 55.5 53.0 55.0 57.0 48.5 57.0 | 110110000000 | 345 | 345 | 11, 48, 19, 48, 26, 48, 26 |
| **2007** | SZ | clinical | 07278-2 | 63.5 63.0 61.5 53.0 62.0 65.0 55.5 53.0 55.0 57.0 48.5 57.0 | 110110000000 | 345 | 345 | 11, 48, 19, 48, 26, 48, 26 |
| **2008** | SZ | clinical | 08001 | 63.5 63.0 61.5 53.0 62.5 65.0 55.0 53.0 55.0 56.5 48.5 57.0 | 110110000000 | 345 | 345 | 11, 48, 19, 48, 26, 48, 26 |
| **2008** | SZ | clinical | 08147 | 63.5 63.0 61.5 53.0 62.5 65.0 55.0 53.0 55.0 56.5 48.5 57.0 | 110110000000 | 345 | 345 | 11, 48, 19, 48, 26, 48, 26 |
| **2008** | SZ | clinical | 08018 | 63.5 63.0 61.5 52.5 62.5 65.0 55.0 53.0 54.5 56.5 48.5 57.0 | 110110000000 | 345 | **962** | 11, 48, 112, 48, 26, 48, 26 |
| **2011** | SZ | clinical | 11084 | 63.5 63.0 61.0 53.0 62.5 65.0 55.0 52.5 54.5 56.5 48.5 57.0 | 110110000000 | 345 | 189 | 11, 48, 3, 48, 26, 48, 26 |
| **2011** | SZ | clinical | 11093 | 63.5 63.0 61.0 53.0 62.0 65.0 55.0 52.5 54.5 56.5 48.5 57.0 | 110110000000 | 345 | 189 | 11, 48, 3, 48, 26, 48, 26 |
| **2011** | SZ | clinical | 11005 | 64.0 63.5 61.5 53.0 62.5 65.0 55.0 53.0 54.5 56.5 48.5 57.0 | 110110000000 | 345 | 265 | 11, 48, 107, 48, 26, 48, 26 |
| **2011** | SZ | clinical | 11102 | 64.0 63.5 61.5 53.0 62.5 65.0 55.0 52.5 54.5 56.5 48.5 57.0 | 110110000000 | 345 | 265 | 11, 48, 107, 48, 26, 48, 26 |
| **2011** | SZ | clinical | 11107 | 63.5 63.0 61.0 53.0 62.5 65.0 55.0 52.5 54.5 56.5 48.5 57.0 | 110110000000 | 345 | 265 | 11, 48, 107, 48, 26, 48, 26 |
| **2011** | SZ | clinical | 11109 | 63.5 63.0 61.0 53.0 62.5 65.0 55.0 52.5 54.5 57.0 48.5 57.0 | 110110000000 | 345 | 265 | 11, 48, 107, 48, 26, 48, 26 |
| **2011** | SZ | clinical | 11110 | 63.5 63.0 61.0 53.0 62.5 65.0 55.0 52.5 54.5 57.0 48.5 57.0 | 110110000000 | 345 | 265 | 11, 48, 107, 48, 26, 48, 26 |
| **2011** | SZ | clinical | 11111 | 63.5 63.0 61.0 53.0 62.5 65.0 55.0 52.5 54.5 57.0 48.5 57.0 | 110110000000 | 345 | 265 | 11, 48, 107, 48, 26, 48, 26 |
| **2011** | SZ | clinical | 11213 | 63.5 63.0 61.0 53.0 62.5 65.0 55.0 52.5 54.5 56.5 48.5 57.0 | 110110000000 | 345 | 265 | 11, 48, 107, 48, 26, 48, 26 |
| **2011** | SZ | clinical | 11237 | 63.5 63.0 61.5 53.0 62.5 65.0 55.0 52.5 54.5 56.5 48.5 57.0 | 110110000000 | 345 | 265 | 11, 48, 107, 48, 26, 48, 26 |
| **2011** | SZ | clinical | 11032 | 63.5 63.0 61.0 53.0 62.0 65.0 55.0 53.0 54.5 56.5 48.5 57.0 | 110110000000 | 345 | 345 | 11, 48, 19, 48, 26, 48, 26 |
| **2011** | SZ | clinical | 11185 | 63.5 63.0 61.5 53.0 62.5 65.0 55.0 52.5 54.5 56.5 48.5 57.0 | 110110000000 | 345 | 345 | 11, 48, 19, 48, 26, 48, 26 |
| **2011** | SZ | clinical | 11228 | 63.5 59.0 68.5 53.0 55.5 65.0 55.0 52.0 54.5 56.5 48.5 57.0 | 101100000000 | 497 | **961** | 5, 137, 125, 50, 23, 196, 79 |
| **2007** | SZ | clinical | 07265 | 60.5 53.0 61.0 49.5 55.5 65.0 63.0 53.0 54.5 57.0 48.5 57.0 | 000000100000 | 527 | 527 | 43, 41, 107, 42, 37, 40, 33 |
| **2007** | SZ | clinical | 07266 | 61.0 52.5 61.0 50.0 55.5 65.0 63.0 53.0 54.5 56.5 48.5 57.0 | 000000100000 | 527 | 527 | 43, 41, 107, 42, 37, 40, 33 |
| **2007** | SZ | clinical | 07268 | 61.0 53.0 61.0 50.0 55.5 65.0 63.0 53.0 54.5 56.5 48.5 57.0 | 000000100000 | 527 | 527 | 43, 41, 107, 42, 37, 40, 33 |
| **2011** | SZ | clinical | 11036 | 60.5 53.0 61.0 50.0 55.5 65.0 63.0 53.0 54.5 56.5 48.5 56.5 | 000000100000 | 527 | **960** | 43, 41, 27, 42, 37, 40, 33 |
| **2007** | SZ | clinical | 07003 | 60.5 63.0 61.0 50.5 55.5 65.0 63.0 53.0 54.0 60.5 48.5 56.5 | 010000100100 | 781 | **959** | 31, 371, 67, 13, 4, 299, 1 |
| **2005** | XM | clinical | 33 | 60.5 63.0 61.0 52.5 64.0 64.5 55.0 59.0 54.5 56.0 55.5 61.5 | 010110010011 | 3 | 3 | 3, 4, 19, 4, 29, 4, 22 |
| **2006** | XM | clinical | 42 | 60.5 63.0 61.0 52.5 64.0 64.5 55.0 59.0 54.5 56.0 55.5 61.5 | 010110010011 | 3 | 3 | 3, 4, 19, 4, 29, 4, 22 |
| **2006** | XM | clinical | 60 | 60.5 63.0 61.0 52.5 64.0 64.5 55.0 58.5 54.5 56.0 55.5 61.5 | 010110010011 | 3 | 3 | 3, 4, 19, 4, 29, 4, 22 |
| **2006** | XM | clinical | 75 | 60.5 63.0 61.0 52.5 64.0 64.5 54.5 58.5 54.5 56.0 55.0 61.5 | 010110010011 | 3 | 3 | 3, 4, 19, 4, 29, 4, 22 |
| **2008** | XM | clinical | 123 | 60.5 63.0 61.0 52.5 64.0 64.5 55.0 58.5 54.5 56.0 55.5 61.5 | 010110010011 | 3 | 3 | 3, 4, 19, 4, 29, 4, 22 |
| **2011** | XM | clinical | 2011024 | 60.5 62.5 60.5 52.5 63.5 64.0 55.0 58.5 54.5 56.0 55.5 61.5 | 010110010011 | 3 | 3 | 3, 4, 19, 4, 29, 4, 22 |
| **2011** | XM | clinical | 2011026 | 60.5 62.5 60.5 52.5 63.5 64.0 54.5 58.5 54.5 56.0 55.5 61.5 | 010110010011 | 3 | 3 | 3, 4, 19, 4, 29, 4, 22 |
| **2011** | XM | clinical | 2011029 | 60.5 63.0 60.5 52.5 63.5 64.0 55.0 59.0 54.5 56.0 55.5 61.5 | 010110010011 | 3 | 3 | 3, 4, 19, 4, 29, 4, 22 |
| **2011** | XM | clinical | 2011032 | 60.5 63.0 60.5 52.5 63.5 64.0 55.0 59.0 54.5 56.0 55.5 61.5 | 010110010011 | 3 | 3 | 3, 4, 19, 4, 29, 4, 22 |
| **2011** | XM | clinical | 2011035 | 60.5 63.0 60.5 52.5 63.5 64.0 55.0 59.0 54.5 56.0 55.5 61.5 | 010110010011 | 3 | 3 | 3, 4, 19, 4, 29, 4, 22 |
| **2011** | XM | clinical | 2011038 | 60.5 63.0 60.5 52.5 63.5 64.0 55.0 59.0 54.5 56.0 55.5 61.5 | 010110010011 | 3 | 3 | 3, 4, 19, 4, 29, 4, 22 |
| **2005** | XM | clinical | 31 | 61.0 63.5 60.5 53.0 64.5 65.0 55.0 59.0 54.5 56.0 55.5 61.0 | 010110010011 | 3 | 3 | 3, 4, 19, 4, 29, 4, 22 |
| **2005** | XM | clinical | 32 | 61.0 63.5 60.5 53.0 64.5 65.0 55.0 59.0 54.5 56.0 55.5 61.0 | 010110010011 | 3 | 3 | 3, 4, 19, 4, 29, 4, 22 |
| **2005** | XM | clinical | 34 | 61.0 63.5 60.5 53.0 64.5 65.0 55.0 59.0 54.5 56.0 55.5 61.0 | 010110010011 | 3 | 3 | 3, 4, 19, 4, 29, 4, 22 |
| **2005** | XM | clinical | 35 | 61.0 63.5 60.5 53.0 64.5 65.0 55.0 59.0 54.5 56.0 55.5 61.0 | 010110010011 | 3 | 3 | 3, 4, 19, 4, 29, 4, 22 |
| **2006** | XM | clinical | 43 | 61.0 63.5 60.5 53.0 64.5 65.0 55.0 59.0 54.5 56.0 55.5 61.0 | 010110010011 | 3 | 3 | 3, 4, 19, 4, 29, 4, 22 |
| **2006** | XM | clinical | 46 | 61.0 63.5 60.5 53.0 64.5 65.0 55.0 59.0 54.5 56.0 55.5 61.0 | 010110010011 | 3 | 3 | 3, 4, 19, 4, 29, 4, 22 |
| **2006** | XM | clinical | 61 | 61.0 63.5 60.5 53.0 64.5 65.0 55.0 59.0 54.5 56.0 55.5 61.0 | 010110010011 | 3 | 3 | 3, 4, 19, 4, 29, 4, 22 |
| **2006** | XM | clinical | 72 | 61.0 63.5 60.5 53.0 64.5 65.0 55.0 59.0 54.5 56.0 55.5 61.0 | 010110010011 | 3 | 3 | 3, 4, 19, 4, 29, 4, 22 |
| **2006** | XM | clinical | 74 | 61.0 63.5 60.5 53.0 64.5 65.0 55.0 59.0 54.5 56.0 55.5 61.0 | 010110010011 | 3 | 3 | 3, 4, 19, 4, 29, 4, 22 |
| **2006** | XM | clinical | 77 | 61.0 63.5 60.5 53.0 64.5 65.0 55.0 59.0 54.5 56.0 55.5 61.0 | 010110010011 | 3 | 3 | 3, 4, 19, 4, 29, 4, 22 |
| **2008** | XM | clinical | 125 | 61.0 63.5 60.5 53.0 64.5 65.0 55.0 59.0 54.5 56.0 55.5 61.0 | 010110010011 | 3 | 3 | 3, 4, 19, 4, 29, 4, 22 |
| **2010** | XM | clinical | 175 | 61.0 63.5 60.5 53.0 64.5 65.0 55.0 59.0 54.5 56.0 55.5 61.0 | 010110010011 | 3 | 3 | 3, 4, 19, 4, 29, 4, 22 |
| **2010** | XM | clinical | 179 | 61.0 63.5 60.5 53.0 64.5 65.0 55.0 59.0 54.5 56.0 55.5 61.0 | 010110010011 | 3 | 3 | 3, 4, 19, 4, 29, 4, 22 |
| **2010** | XM | clinical | 180 | 61.0 63.5 60.5 53.0 64.5 65.0 55.0 59.0 54.5 56.0 55.5 61.0 | 010110010011 | 3 | 3 | 3, 4, 19, 4, 29, 4, 22 |
| **2010** | XM | clinical | 181 | 61.0 63.5 60.5 53.0 64.5 65.0 55.0 59.0 54.5 56.0 55.5 61.0 | 010110010011 | 3 | 3 | 3, 4, 19, 4, 29, 4, 22 |
| **2010** | XM | clinical | 182 | 61.0 63.5 60.5 53.0 64.5 65.0 55.0 59.0 54.5 56.0 55.5 61.0 | 010110010011 | 3 | 3 | 3, 4, 19, 4, 29, 4, 22 |
| **2011** | XM | clinical | 2011025 | 61.0 63.5 60.5 53.0 64.5 65.0 55.0 59.0 54.5 56.0 55.5 61.0 | 010110010011 | 3 | 3 | 3, 4, 19, 4, 29, 4, 22 |
| **2011** | XM | clinical | 2011027 | 61.0 63.5 60.5 53.0 64.5 65.0 55.0 59.0 54.5 56.0 55.5 61.0 | 010110010011 | 3 | 3 | 3, 4, 19, 4, 29, 4, 22 |
| **2011** | XM | clinical | 2011028 | 61.0 63.5 60.5 53.0 64.5 65.0 55.0 59.0 54.5 56.0 55.5 61.0 | 010110010011 | 3 | 3 | 3, 4, 19, 4, 29, 4, 22 |
| **2011** | XM | clinical | 2011031 | 61.0 63.5 60.5 53.0 64.5 65.0 55.0 59.0 54.5 56.0 55.5 61.0 | 010110010011 | 3 | 3 | 3, 4, 19, 4, 29, 4, 22 |
| **2011** | XM | clinical | 2011033 | 61.0 63.5 60.5 53.0 64.5 65.0 55.0 59.0 54.5 56.0 55.5 61.0 | 010110010011 | 3 | 3 | 3, 4, 19, 4, 29, 4, 22 |
| **2006** | XM | clinical | 93 | 60.5 63.0 68.5 49.5 64.0 67.0 45.5 52.0 54.0 56.0 55.5 61.5 | 011011000011 | 8 | 937 | 28, 4, 64, 88, 63, 69, 26 |
| **2010** | XM | clinical | 183 | 60.5 63.0 68.5 49.5 64.0 66.5 46.0 52.5 54.0 56.0 55.5 61.5 | 011011000011 | 8 | 8 | 28, 4, 82, 88, 63, 69, 1 |
| **2010** | XM | uncooked oyster | 231 | 60.5 63.0 68.5 52.0 62.0 66.5 54.5 53.0 57.5 56.0 48.5 61.5 | 011111001001 | 16 | 16 | 12, 13, 15, 22, 3, 1, 12 |
| **2010** | XM | uncooked oyster | 189 | 63.5 63.0 68.5 49.5 55.0 64.5 55.0 52.5 54.0 56.0 48.5 57.0 | 111000000000 | 28 | **949** | 31, 336, 264, 339, 26, 45, 24 |
| **2010** | XM | fresh fish | 164 | 63.5 63.0 68.5 49.5 55.0 67.0 55.0 52.5 54.0 56.0 48.5 50.5 | 111001000000 | 32 | **944** | 111, 17, 262, 123, 85, 37, 87 |
| **2008** | XM | cooked crab | 131 | 63.5 63.0 61.5 50.0 55.5 67.0 62.5 52.5 54.0 42.5 48.5 57.0 | 110001100000 | 34 | **964** | 170, 224, 75, 139, 50, 18, 124 |
| **2011** | XM | uncooked oyster | 2011011 | 63.0 62.5 61.0 49.0 62.0 64.0 55.0 52.5 54.5 56.0 55.5 57.0 | 110010000010 | 61 | 114 | 55, 15, 31, 55, 18, 58, 46 |
| **2011** | XM | clinical | 2011037 | 60.5 63.0 68.5 52.0 62.0 64.0 55.0 53.0 54.5 56.0 55.5 61.5 | 011110000011 | 147 | 170 | 82, 85, 78, 19, 60, 69, 24 |
| **2009** | XM | fresh fish | 134 | 63.0 63.0 68.5 49.5 55.5 64.5 55.5 52.5 54.0 60.0 48.5 57.0 | 111000000100 | 162 | **943** | 265, 104, 261, 41, 34, 233, 41 |
| **2010** | XM | seawater | 241 | 63.0 62.5 68.5 49.0 62.0 64.0 62.5 52.5 54.0 56.0 48.5 61.5 | 111010100001 | 177 | 846 | 60, 354, 250, 305, 26, 54, 84 |
| **2010** | XM | seawater | 242 | 63.0 58.5 68.5 49.5 54.5 64.0 62.5 52.5 54.5 56.5 48.5 57.0 | 101000100000 | 185 | **955** | 264, 364, 67, 330, 184, 11, 132 |
| **2008** | XM | cooked fish | 130 | 63.5 63.0 68.5 52.5 55.5 64.5 62.5 52.5 54.5 56.0 55.5 61.5 | 111100100011 | 191 | **941** | 44, 372, 61, 90, 26, 262, 12 |
| **2010** | XM | clinical | 171 | 60.5 63.0 61.5 56.5 55.0 67.0 62.5 52.0 54.0 57.0 48.5 56.5 | 010101100000 | 199 | 199 | 22, 28, 17, 13, 8, 19, 14 |
| **2010** | XM | clinical | 172 | 60.5 63.0 61.0 56.0 54.5 66.5 62.5 52.0 54.0 56.5 48.5 56.5 | 010101100000 | 199 | 199 | 22, 28, 17, 13, 8, 19, 14 |
| **2010** | XM | clinical | 174 | 60.5 63.0 61.0 56.0 54.5 66.5 62.5 52.0 54.0 56.5 48.5 57.0 | 010101100000 | 199 | 199 | 22, 28, 17, 13, 8, 19, 14 |
| **2010** | XM | seawater | 228 | 60.5 63.0 71.5 52.5 54.5 66.5 53.5 52.5 54.5 56.0 48.5 50.5 | 011101000000 | 212 | 212 | 69, 92, 69, 114, 54, 71, 24 |
| **2007** | XM | clinical | 109 | 63.5 63.0 68.5 52.0 62.0 67.0 62.5 52.5 54.5 60.0 48.5 56.5 | 111111100100 | 216 | 216 | 98, 135, 112, 107, 77, 97, 26 |
| **2007** | XM | clinical | 110 | 63.5 63.0 69.0 52.0 62.0 67.0 62.5 53.0 54.5 60.0 48.5 57.0 | 111111100100 | 216 | 216 | 98, 135, 112, 107, 77, 97, 26 |
| **2007** | XM | clinical | 111 | 63.5 63.0 68.5 52.0 62.0 67.0 62.5 53.0 54.5 60.0 48.5 57.0 | 111111100100 | 216 | 216 | 98, 135, 112, 107, 77, 97, 26 |
| **2010** | XM | fresh fish | 184 | 63.5 63.0 64.0 52.0 55.0 64.5 55.5 52.5 54.5 56.0 48.5 57.0 | 110100000000 | 251 | **945** | 266, 316, 263, 337, 150, 11, 62 |
| **2010** | XM | uncooked oyster | 246 | 63.0 62.5 68.5 49.0 54.5 64.0 62.5 47.5 58.0 56.5 48.5 56.5 | 111000101000 | 338 | **1023** | 36, 343, 31, 76, 98, 11, 84 |
| **2007** | XM | clinical | 112 | 63.5 63.0 61.5 52.5 62.0 64.5 55.0 53.0 54.5 56.5 48.5 57.0 | 110110000000 | 345 | 189 | 11, 48, 3, 48, 26, 48, 26 |
| **2007** | XM | clinical | 113 | 63.5 63.0 61.5 52.0 62.0 64.5 55.0 53.0 54.5 56.5 48.5 57.0 | 110110000000 | 345 | 265 | 11, 48, 107, 48, 26, 48, 26 |
| **2008** | XM | clinical | 129 | 63.5 63.0 61.5 52.0 62.0 64.5 55.0 52.5 54.5 56.5 48.5 57.0 | 110110000000 | 345 | 265 | 11, 48, 107, 48, 26, 48, 26 |
| **2010** | XM | uncooked oyster | 243 | 63.0 62.5 68.5 49.0 62.0 64.0 54.5 47.5 58.0 56.5 55.5 61.5 | 111010001011 | 352 | **1022** | 36, 188, 146, 164, 26, 3, 1 |
| **2011** | XM | uncooked oyster | 2011009 | 63.0 62.5 61.0 49.5 54.5 66.5 54.5 52.0 54.0 60.0 48.5 56.5 | 110001000100 | 370 | **958** | 268, 123, 265, 98, 18, 303, 54 |
| **2010** | XM | uncooked oyster | 229 | 60.5 52.5 68.5 53.0 54.5 64.0 55.0 52.5 54.0 56.0 55.5 57.0 | 001100000010 | 379 | **965** | 158, 23, 65, 74, 66, 154, 33 |
| **2010** | XM | seawater | 230 | 63.0 63.0 68.5 52.0 54.5 66.5 55.0 53.0 57.5 56.5 55.5 56.5 | 111101001010 | 400 | 400 | 12, 208, 159, 133, 28, 128, 86 |
| **2011** | XM | uncooked oyster | 2011006 | 63.0 62.5 68.5 49.5 62.0 66.5 62.5 52.0 54.0 56.0 55.5 61.5 | 111011100011 | 473 | 829 | 28, 106, 82, 27, 18, 69, 26 |
| **2010** | XM | uncooked oyster | 219 | 60.5 52.5 61.0 49.5 54.5 64.0 62.5 52.5 54.0 56.5 48.5 57.0 | 000000100000 | 527 | **1018** | 31, 343, 207, 201, 144, 7, 108 |
| **2010** | XM | uncooked oyster | 240 | 60.5 63.0 61.0 49.0 54.5 66.5 55.0 52.0 54.5 60.0 48.5 56.5 | 010001000100 | 549 | 859 | 208, 300, 213, 29, 147, 46, 24 |
| **2011** | XM | seawater | 2011002 | 55.0 52.5 61.0 52.5 62.0 66.5 55.0 53.0 58.0 56.0 48.5 61.5 | 000111001001 | 635 | **956** | 48, 5, 260, 261, 140, 7, 108 |
| **2010** | XM | uncooked oyster | 236 | 60.5 63.0 68.5 52.5 54.5 64.0 55.0 53.0 54.5 56.5 55.5 56.5 | 011100000010 | 644 | **1021** | 82, 387, 278, 19, 60, 69, 24 |
| **2011** | XM | clinical | 2011034 | 60.5 63.0 61.0 52.0 62.0 64.0 54.5 52.5 54.5 56.5 55.5 57.0 | 010110000010 | 672 | **1034** | 132, 391, 31, 29, 197, 314, 26 |
| **2010** | XM | fresh crab | 188 | 60.5 63.0 68.5 52.5 55.0 67.0 52.0 52.0 54.5 60.0 48.5 56.5 | 011101000100 | 868 | **948** | 51, 104, 3, 338, 46, 45, 24 |
| **2006** | XM | uncooked fish | 66 | 63.5 63.0 68.5 49.5 62.0 64.5 55.0 52.5 54.0 56.0 48.5 61.5 | 111010000001 | 903 | 903 | 31, 312, 25, 313, 26, 247, 24 |
| **2009** | XM | fresh fish | 133 | 55.5 63.0 61.5 53.0 55.5 67.5 62.5 52.0 54.0 42.5 55.5 57.0 | 010101100010 | 919 | 919 | 93, 224, 75, 139, 117, 223, 124 |
| **2006** | XM | uncooked oyster | 56 | 60.5 63.0 68.5 49.5 64.0 64.5 62.5 52.5 54.0 56.0 48.5 61.5 | 011010100001 | 940 | **940** | 272, 4, 25, 108, 190, 263, 86 |
| **2008** | XM | cooked octopus | 132 | 63.0 63.0 61.5 52.0 55.5 64.5 62.5 52.5 54.5 56.5 48.5 57.0 | 110100100000 | 942 | **942** | 42, 117, 31, 13, 187, 46, 23 |
| **2010** | XM | fresh fish | 185 | 60.5 63.0 68.5 52.0 55.0 64.0 62.5 52.5 54.5 60.0 55.0 57.0 | 011100100110 | 946 | **946** | 42, 1, 80, 27, 60, 300, 20 |
| **2010** | XM | fresh fish | 187 | 60.5 63.0 61.5 52.5 45.0 64.5 62.5 58.5 54.5 56.0 48.5 61.5 | 010100110001 | 947 | **947** | 133, 373, 71, 170, 189, 74, 86 |
| **2010** | XM | uncooked oyster | 197 | 63.0 63.0 61.0 52.5 45.0 64.0 54.5 58.5 54.5 56.0 55.0 57.0 | 110100010010 | 950 | **950** | 3, 374, 31, 214, 26, 119, 54 |
| **2010** | XM | seawater | 202 | 60.0 52.5 61.0 52.0 62.0 64.0 55.0 52.5 54.5 56.0 48.5 61.5 | 000110000001 | 951 | **951** | 42, 153, 61, 340, 56, 17, 9 |
| **2010** | XM | soil | 209 | 63.0 58.5 61.0 52.5 54.5 64.0 54.5 58.5 57.5 56.5 55.5 56.5 | 100100011010 | 952 | **952** | 269, 25, 266, 280, 50, 78, 94 |
| **2010** | XM | uncooked oyster | 218 | 60.5 52.5 68.5 49.0 54.5 64.0 62.5 47.5 58.0 56.0 49.0 57.0 | 001000101000 | 953 | **953** | 270, 51, 61, 341, 4, 301, 37 |
| **2010** | XM | seawater | 238 | 63.0 63.0 61.0 53.0 63.5 64.0 62.5 52.5 54.0 56.0 48.5 61.5 | 110110100001 | 954 | **954** | 271, 4, 267, 170, 188, 302, 2 |
| **2011** | XM | uncooked oyster | 2011005 | 63.0 62.5 68.0 49.5 54.5 64.0 55.0 59.0 54.0 56.0 55.5 61.5 | 111000010011 | 957 | **957** | 267, 372, 61, 181, 21, 262, 12 |
| **2006** | XM | clinical | 95 | 63.5 63.0 61.5 52.5 55.0 67.0 62.5 52.5 54.5 56.5 55.5 56.5 | 110101100010 | 963 | **963** | 51, 25, 30, 314, 23, 78, 26 |
| **2010** | XM | seawater | 225 | 60.5 63.0 68.5 53.0 54.5 64.0 55.0 52.5 54.0 42.5 55.5 61.5 | 011100000011 | 1019 | **1019** | 158, 261, 226, 299, 2, 192, 54 |
| **2010** | XM | seawater | 233 | 60.5 63.0 68.5 49.0 63.5 66.5 55.0 53.0 58.0 56.0 48.5 61.5 | 011011001001 | 1020 | **1020** | 175, 4, 277, 344, 49, 311, 26 |
| **2010** | XM | uncooked oyster | 217 | 60.5 63.0 61.0 52.5 63.5 64.0 62.5 59.0 54.5 56.0 49.0 57.0 | 010110110000 | 1033 | **1033** | 3, 390, 280, 175, 31, 39, 26 |

^a^XM, Xiamen; SZ, Shenzhen.

^b^ The new STs found in this study are given in bold letters.

^c^The order of the loci is *dnaE, gyrB, recA,dtdS, pntA, pyrC, tnaA.*
